# Tunnelling Nanotube‐Mediated Lysosome Sharing Promotes Osteocyte Survival via Transcellular Autophagy

**DOI:** 10.1111/cpr.70226

**Published:** 2026-05-14

**Authors:** Jinbiao Qiang, Ronghao Jin, Tong Sha, Fang Zheng, Yijun Zhou, Yue Hu, Shuyu Zhang, Zhenming Yang, Mengdong Nie, Huanyu Luo, Xiaoduo Tang, Hao Guo, Zunxuan Xie, Jinwei Li, Hongchen Sun, Cangwei Liu, Ce Shi

**Affiliations:** ^1^ Department of Oral Pathology, Hospital of Stomatology Jilin University Changchun China; ^2^ Jilin Provincial Key Laboratory of Oral and Craniofacial Diseases & Tissue Reconstruction Changchun China; ^3^ Key Laboratory of Pathobiology, Ministry of Education Jilin University Changchun China; ^4^ Department of Oral and Maxillofacial Surgery, Hospital of Stomatology Jilin University Changchun China; ^5^ Department of Endodontics, Hospital of Stomatology Jilin University Changchun China; ^6^ School and Hospital of Stomatology, China Medical University Shenyang China; ^7^ Department of Stomatology Puyang Oilfield General Hospital Puyang China; ^8^ Department of Stomatology Puyang People's Hospital Puyang China

**Keywords:** apoptosis, intercellular communication, lysosomes, mitochondria, osteocytes, transcellular autophagy, tunnelling nanotubes

## Abstract

Osteocytes, the central regulators of bone remodelling, are essential for maintaining bone homeostasis. Embedded in a nutrient‐limited matrix and burdened by cumulative stress over their exceptionally long lifespan, how osteocytes sustain long‐term viability remains elusive. Tunnelling nanotubes (TNTs) are newly described intercellular bridges that enable long‐range transfer of organelles and have been implicated in stress adaptation. Here, we provide the first definitive identification of TNTs between cultured osteocytes, which exhibit canonical TNT morphology together with osteocyte‐specific features. Functionally, osteocytic TNTs mediate intercellular transfer of membrane‐bound cargo, predominantly lysosomes. Under nutrient deprivation, TNT formation and lysosome transfer are both increased, replenishing the lysosomal pool in stressed osteocytes. Transferred lysosomes then fuse with accumulated autophagosomes, thereby restoring impaired autophagic flux and suppressing apoptosis. This cytoprotective effect requires TNT integrity and intact autophagic flux. Although mitochondrial transfer is detectable, it does not confer comparable protection. The findings identify a transcellular autophagy pathway mediated by TNT‐dependent lysosome sharing, revealing a previously unrecognized cooperative survival strategy among osteocytes. This work establishes a novel conceptual framework in osteocyte biology and suggests potential therapeutic avenues for bone diseases associated with osteocyte apoptosis and impaired bone remodelling.

## Graphical abstract

This study provides the first definitive identification of tunnelling nanotubes (TNTs) between cultured osteocytes and shows TNT‐mediated lysosome transfer from healthy to stressed cells, restoring autophagic flux and preventing apoptosis. This discovery of transcellular autophagy reveals a new survival mechanism in osteocytes, offering potential therapeutic targets for bone diseases.

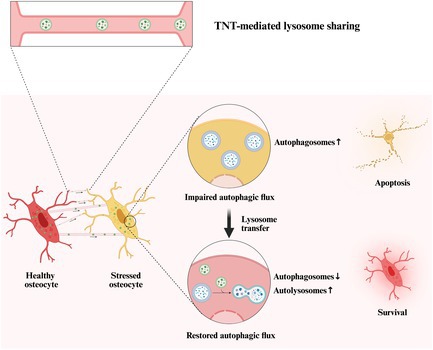

## 1 Introduction

Bone is essential for organismal function, and its structural and functional integrity is maintained through continuous bone remodelling [1, 2]. Osteocytes, comprising ~90%–95% of bone cells, orchestrate bone remodelling and are indispensable for maintaining bone homeostasis [3, 4]. However, osteocytes reside deep within the mineralized matrix and communicate via the lacunar–canalicular system (LCS), where nutrient availability is limited [5]. As terminally differentiated cells, they exhibit minimal proliferative capacity and cannot be readily replaced by newly differentiated cells [5]. Moreover, osteocytes can survive for decades, exposing them to cumulative physiological and pathological stresses, including mechanical loading, bone microdamage and hormonal fluctuations, which can ultimately drive predominantly apoptotic cell death [6, 7]. Osteocyte death and loss are considered key contributors to a range of bone‐related disorders, including osteoporosis [8]. These observations suggest that osteocytes have likely evolved unique protective mechanisms to preserve long‐term viability within this microenvironment [9]. Nevertheless, currently recognized osteocyte‐intrinsic survival pathways may be insufficient to explain how osteocytes withstand persistent stress [8]. Therefore, elucidating specialized strategies that sustain osteocyte survival is of great significance for understanding bone homeostasis and its disruption in disease.

Intercellular communication is a prerequisite for the survival of multicellular organisms [10]. This communication can be generally categorized into indirect (e.g., paracrine) and direct modes [11]. Of these, direct intercellular communication facilitates expeditious and precise signal transmission, thereby ensuring coordinated cellular responses within cell populations and tissues. This process is of paramount importance for maintaining normal physiological functions [10, 11, 12]. Osteocytes have been demonstrated to engage in direct communication via gap junctional intercellular communication [13, 14]. However, gap junction channels are incapable of transmitting large macromolecules, such as organelles, which are often indispensable for survival signalling [15, 16]. Therefore, the existence of additional types of direct communication within the osteocytic network remains to be fully elucidated. Tunnelling nanotubes (TNTs) are F‐actin‐based tubular structures that enable direct intercellular communication between two or more cells, representing a novel mechanism of cell‐to‐cell interaction identified in recent years [17]. These dynamic structures mediate the intercellular transfer of diverse cargoes, including proteins, vesicles and organelles, thereby enabling the transfer of both signalling and metabolic components and implicating TNTs in a wide range of biological and pathological processes [18, 19, 20]. Unlike gap junctions, TNTs are capable of transferring not only small molecules but also macromolecular cargoes, and they can bridge long distances, ranging from several to hundreds of micrometres between cells. Increasing evidence indicates that TNTs serve as pivotal conduits for cellular survival under stress conditions, thereby enhancing the resilience of compromised or damaged cells [19, 21, 22, 23].

To date, TNTs have been identified in various cell types, including tumour cells [24], immune cells [25], stem cells [26] and neurons [22, 27, 28, 29]. In bone tissue‐related cell populations, such as osteoclasts and their precursors [30], bone marrow mesenchymal stem cells (BMSCs) [18, 31], and osteoblasts [32], the presence of TNTs has also been successively documented. However, direct evidence confirming the existence of bona fide TNTs between osteocytes remains lacking. Several observations lead us to hypothesize that TNTs may indeed form between osteocytes. Morphologically, osteocytes share notable similarities with known TNT‐forming cells such as dendritic cells and neurons [22, 25, 27, 28, 29, 33], including an elongated dendritic architecture conducive to nanotube formation. Within bone tissues, the extensive interconnectivity of osteocytes via the LCS and the maintenance of specific spatial distances between adjacent cells further fulfil the key structural prerequisites for TNT formation. In addition, given the long lifespan of osteocytes, their continual exposure to diverse stresses, and the established function of TNTs in rescuing damaged cells under stress [19, 21, 22], it is plausible that osteocytes utilize TNT‐mediated communication to transfer essential survival signals that support their adaptation and viability within the nutrient‐limited bone microenvironment.

In this study, we report, for the first time, the presence of TNTs connecting osteocytes and systematically characterize their osteocytic specificity. Of particular significance, we demonstrate that healthy osteocytes transfer lysosomes to stressed osteocytes via these TNTs. The transferred lysosomes effectively fuse with accumulated autophagosomes in the recipient cells, thereby significantly restoring impaired autophagic flux, inhibiting apoptosis and promoting osteocyte survival. Our findings unveil a previously unrecognized lysosome‐sharing mechanism facilitated by TNTs and identify a novel form of transcellular autophagy between osteocytes. This study provides a new conceptual framework for understanding the structural and functional organization of the osteocytic communication network and opens new avenues for developing interventions for bone diseases (e.g., osteoporosis and osteonecrosis) by targeting the TNT–lysosome–autophagy–survival axis.

## 2 Materials and Methods

### 2.1 Ethics Statement

All animal experiments were reviewed and approved by the Ethics Review Committee and the Animal Care and Use Committee of the Basic Medical College of Jilin University (Approval Number: 2025‐559). Human jawbone samples were obtained from the Hospital of Stomatology of Jilin University, with approval from the Medical Ethics Committee of the Hospital of Stomatology of Jilin University (Approval Number: SJDKQ2025101). All participants signed informed consent before sample collection during impacted tooth extraction surgery. All procedures were conducted in accordance with the institutional guidelines and the Declaration of Helsinki.

### 2.2 Cell Culture

MLO‐Y4 murine osteocyte‐like cells were obtained from the Hospital of Stomatology, Jilin University. Cells were cultured in α‐MEM (C12571500BT, Gibco, USA) supplemented with fetal bovine serum (FBS, 10%, BS‐1102, Opcel, China) and penicillin–streptomycin (P/S, 1×, SV30010, HyClone, USA) in a humidified incubator at 37°C with 5% CO_2_. Cells were routinely tested negative for mycoplasma and used within 20 passages.

Primary osteocytes were isolated from C57BL/6J mice (Beijing Vital River Laboratory Animal Technology Co. Ltd.) as previously described [34]. Briefly, tibiae and femora from three mice were dissected, cleaned of soft tissue and marrow, and cut into 1–2 mm fragments, followed by sequential digestions using collagenase type I (300 IU mL^−1^; LS004196, Worthington) and EDTA (5 mM, Tianli Chemical, China). Cells from the final (ninth) digestion were collected as primary osteocytes, cultured in complete medium and used within 7 days. Cells from the fifth digestion (osteoblast‐enriched) were collected as controls for osteocyte identification. Human primary osteocytes were isolated from jawbone samples collected during impacted tooth extraction and processed using the same protocol. All primary osteocytes were mycoplasma‐free prior to experiments.

### 2.3 Identification of Primary Osteocytes

Osteocytes were identified by low to absent alkaline phosphatase (ALP) activity and by expression of *E11* and *DMP1* [14, 34, 35]. ALP activity was assessed using an ALP Staining kit (C3206, Beyotime, China). *E11* and *DMP1* mRNA expression was quantified by real‐time quantitative reverse transcription PCR (qRT‐PCR). Total RNA was extracted using RNAiso Plus (9109, Takara, Japan) and quantified using a NanoDrop One spectrophotometer (ThermoFisher, USA). cDNA was synthesized from total RNA using Hifair III 1st Strand cDNA Synthesis SuperMix (11141ES60, Yeasen, China) and qRT‐PCR was performed with Hieff SYBR Green Master Mix (11202ES08, Yeasen, China) using cDNA (25 ng per reaction). Relative gene expression was calculated using the 2^−ΔΔCt^ method, normalized to β‐actin. The primer sequences used are detailed in Table S1.

### 2.4 TNT Labelling

Cells were cultured to ~70% confluence and then fixed following a protocol optimized for TNT preservation [36]. Briefly, a paraformaldehyde‐glutaraldehyde fixative (2%/0.05%) was added directly to the culture medium and incubated for 4 min, replaced with paraformaldehyde‐glutaraldehyde fixative (2%/0.05%) for 15 min, followed by paraformaldehyde (4%) for 15 min. After washing with PBS, cells were permeabilized with Triton X‐100 (0.2%, CT11451, Coolaber, China) for 5 min, stained with phalloidin (1:100, CA1610 or CA1620, Solarbio, China) for 60 min and counterstained with DAPI (C0065, Solarbio, China) for 5 min. Samples were mounted using ProLong Gold antifade mountant (P36930, ThermoFisher, USA) and stored overnight at 4°C.

### 2.5 Immunofluorescence Staining

After fixation and permeabilization as described above, cells were incubated with a blocking reagent (Maxim Biotechnologies, China) for 30 min. Subsequently, cells were incubated overnight at 4°C with either rabbit anti‐α‐tubulin antibody (1:100, 11224‐1‐AP, Proteintech, USA) or rabbit anti‐Connexin 43 antibody (1:200, C6219, Sigma‐Aldrich, Germany). After PBS washing, cells were incubated with FITC‐conjugated goat anti‐rabbit IgG (H + L) (1:200, SA00003‐2, Proteintech, USA) for 2 h. Cells were then stained with phalloidin for 60 min and counterstained with DAPI for 5 min. Finally, samples were mounted and stored at 4°C overnight.

### 2.6 Confocal Laser Scanning Microscope (CLSM) Imaging

For fixed‐cell imaging, samples were imaged using a CLSM (AXR, Nikon, Japan) with 25× and 100× oil‐immersion objectives. Z‐stacks (≥ 15 sections) were acquired with a step size of 0.3 μm at 2048 × 2048 resolution. 3D reconstruction was performed using Imaris Viewer and NIS‐Elements Viewer. TNTs were identified based on the following criteria: (1) connecting at least two cells; (2) positive for F‐actin; and (3) suspended above the culture substrate [37]. Image‐Pro Plus 6.0 or ImageJ was used to measure the length and diameter of TNTs. The number of TNTs was counted, and the percentage of cells with TNTs and the average number of TNTs per cell were calculated.

For live‐cell imaging, cells were imaged in a temperature‐ and CO_2_‐controlled chamber (37°C, 5% CO_2_) using differential interference contrast (DIC) optics on the CLSM. Time‐lapse images were acquired every 1 min with the Perfect Focus System (Nikon) and analysed using NIS‐Elements Viewer and ImageJ to track TNT formation and cargo transport.

### 2.7 Scanning Electron Microscopy (SEM)

Cells were fixed with pre‐cooled glutaraldehyde (2.5%) overnight at 4°C. After PBS washing, cells were dehydrated in graded ethanol solutions (30%, 50%, 70% and 90%) for 15 min each, followed by two changes of ethanol (100%) for 20 min each. The samples were then subjected to critical point drying, sputter‐coated with gold, and examined under a SEM (JSM‐6700F, JEOL, Japan) to visualize TNTs.

### 2.8 Cytochalasin B and Stress Treatments

To investigate the effects of F‐actin on TNT formation, MLO‐Y4 cells were treated for 24 h with cytochalasin B (0, 0.25, 0.5 or 1 μM). To assess the effects of oxidative stress, cells were treated with H₂O₂ (0, 100 or 200 μM) for 2 or 24 h. To evaluate the effects of nutrient deprivation and/or acidic stress, cells were cultured for 24 h in α‐MEM at pH 7.4 or 6.6 with or without FBS (10% or 0%), with P/S (1×). Following the respective treatments, TNT labelling and quantification were performed as described above. Cell viability was assessed using the Cell Counting Kit‐8 (CCK‐8, IV08, Invigentech, USA) according to the manufacturer's protocol and the absorbance was measured at 450 nm using a microplate reader (Synergy H1, BioTek, USA).

### 2.9 Cell Labelling and Transfection

To label intracellular membrane‐bound cargo, DiD (1.5 μM, D7757, ThermoFisher, USA) was prepared in complete medium and incubated with cells for 25 min at 37°C. Cells were washed three times with pre‐warmed complete medium, with each wash followed by a 10‐min incubation at 37°C to promote internalization. For EGFP labelling, the AdCMV‐EGFP adenoviral vector (provided by Prof. Changyu Zheng, National Institute of Dental and Craniofacial Research, NIH, USA) was diluted in serum‐free, P/S‐free α‐MEM at a multiplicity of infection (MOI) of 800. Cells were incubated for 6 h at 37°C, supplemented with an equal volume of α‐MEM containing 20% FBS, and cultured for an additional 18 h. To label the cytoplasm, cells were incubated with CellTrace Red CMTPX (2 μM, 40717ES50, Yeasen, China) for 25 min at 37°C. For lysosome labelling, cells were incubated with LysoTracker Green DND‐26 dye (70 nM, A66438, ThermoFisher, USA) or LysoTracker Deep Red dye (70 nM, L12492, ThermoFisher, USA) for 1–2 h at 37°C. For mitochondria labelling, cells were incubated with MitoTracker Green FM dye (200 nM, A66441, ThermoFisher, USA) or MitoTracker Orange CMTMRos dye (200 nM, A66442, ThermoFisher, USA) for 20 min at 37°C.

### 2.10 Intercellular Cargo Transfer Assays

To observe the intercellular transport of DiD‐labelled membrane‐bound cargo, LysoTracker‐labelled lysosomes or MitoTracker‐labelled mitochondria, the corresponding pre‐labelled cells served as donor cells, whereas EGFP‐transfected or CMTPX‐labelled cells served as recipient cells. To detect the transfer of these cargoes from healthy cells to stressed cells, the donor cells were cultured under normal conditions, while the recipient cells were starved in serum‐free α‐MEM for 24 h.

For direct co‐culture, donor and recipient cells were mixed at a 1:1 ratio and co‐cultured for 16–24 h. Cytochalasin B (1 μM) was used to inhibit TNT formation, and bafilomycin A1 (BafA1; 50 nM, HY‐100558, MCE, USA) was added for the final 4–6 h to block autophagic flux. To assess indirect communication, conditioned medium (CM) or Transwell culture was performed. For CM culture, DiD‐labelled donor cells were cultured for 24 h, after which the culture supernatant was collected and centrifuged at 1000 × *g* for 10 min to remove cells and debris. The resulting CM was added to CMTPX‐labelled recipient cells for 24 h before flow cytometry. For Transwell culture, donor and recipient cells were seeded at equal numbers in the upper and lower chambers, respectively, using 3‐μm (Labselect; 14152) or 1‐μm (Labselect; 14122) inserts. After 24 h, cells were either subjected to TNT labelling or collected for flow cytometry to assess intercellular cargo transfer.

To assess the colocalization of DiD and LysoTracker signals within TNTs, co‐labelled cells were imaged by CLSM, and the images were analysed using the Coloc 2 plugin in ImageJ. To analyse the dynamic relationship between DiD and LysoTracker signals during transport, a kymograph was generated from live‐cell time‐lapse images of the middle region of a TNT using the KymoResliceWide plugin in ImageJ. The kymograph was used to visualize the dynamic trajectories of DiD and LysoTracker signals passing through this region, to extract the corresponding signal intensity profiles, and to perform colocalization analysis.

### 2.11 Annexin V Apoptosis Assay

Apoptosis was assessed by Annexin V‐FITC staining (A5001‐02A‐L, Simu Biotech, China) according to the manufacturer's protocol. For flow cytometry, cells were detached with EDTA‐free trypsin (0.25%, C0205, Beyotime, China) and stained with Annexin V‐FITC staining solution (5 μL per 1 × 10^5^ cells) for 15 min. For CLSM, cells in confocal dishes at ~70% confluence were stained with staining solution (5 μL per confocal dish) for 15 min.

### 2.12 Flow Cytometry

Before flow cytometric acquisition, cells were filtered through a 70 μm strainer (15‐1070, Biologix, China). Samples were acquired using a flow cytometer (MACSQuant Analyzer 16, Miltenyi Biotec, USA) or sorted by fluorescence‐activated cell sorting (FACS) using a BD Influx (BD Biosciences, USA). Data analysis was performed using FlowJo 10.8.1. The analysed parameters included: (1) the percentage of DiD^+^, LysoTracker^+^ or MitoTracker^+^ cells among recipient cells; and (2) the Annexin‐V‐positive rate (i.e., apoptotic rate) within the following recipient subpopulations: DiD^+^ and DiD^−^ cells, LysoTracker^+^ and LysoTracker^−^ cells and MitoTracker^+^ and MitoTracker^−^ cells. During flow sorting, donor cells, as well as LysoTracker^+^, LysoTracker^−^, MitoTracker^+^ and MitoTracker^−^ recipient cells, were collected for subsequent transmission electron microscopy (TEM) or Western blot.

### 2.13 TEM

Sorted cells were fixed overnight at 4°C with pre‐cooled glutaraldehyde (2.5%), then post‐fixed with osmium tetroxide (1%, 012103, Ted Pella Inc., USA) for 1–2 h. Samples were dehydrated through graded ethanol and two changes of 100% acetone, infiltrated with epoxy embedding kit (02660R‐AB, SPI Supplies, USA) via acetone–resin mixtures (1:1, 1:3) followed by 100% resin, and polymerized. Ultrathin sections (70–90 nm) prepared with an ultramicrotome (UC7, Leica, Germany) were collected on copper grids, stained with uranyl acetate and lead citrate and air‐dried. The cellular ultrastructure was imaged by TEM (HT7800, Hitachi, Japan). Cytoplasmic area and the numbers of lysosomes, autophagosomes and autolysosomes were quantified in ImageJ and expressed as the organelle numbers per 100 μm^2^ of cytoplasm.

### 2.14 Western Blot

Sorted cells were collected and lysed using RIPA lysis buffer (P0013K, Beyotime, China) supplemented with phosphatase inhibitor (HY‐K0021, MCE, USA) and protease inhibitor (HY‐K0010, MCE, USA). Equal amounts of protein were separated by 10%–15% sodium dodecyl sulfate‐polyacrylamide gel electrophoresis and transferred onto polyvinylidene fluoride membranes. The membranes were blocked with Rapid Blocking Buffer (PS108P, Epizyme Biotech, China) and incubated overnight at 4°C with rabbit anti‐p62 (1:1000, A0964, ABclonal, China), rabbit anti‐LC3B (1:1000, A19665, ABclonal, China) and mouse anti‐β‐actin (1:50000, 66009‐1‐Ig, Proteintech, USA). After TBST washes, membranes were incubated with HRP‐conjugated goat anti‐rabbit IgG (1:10000, SA00001‐2, Proteintech, USA) or HRP‐conjugated goat anti‐mouse IgG (1:10000, SA00001‐1, Proteintech, USA) for 2 h. Protein bands were visualized by an enhanced chemiluminescence detection kit (PK10001, Proteintech, USA) and signals were captured using a multifunctional gel imaging analysis system (5200, Tanon, China). Band intensity was quantified using ImageJ.

### 2.15 3D Culture of Osteocytes

MLO‐Y4 cells labelled with DiD were resuspended in 5× α‐MEM at a density of 0.5 × 10^4^ cells per mL. Pre‐cooled Cellmatrix Type I‐A (35 μL, 637‐00653, Nitta Gelatin, Japan) was mixed with cell suspension (10 μL in 5× α‐MEM) and Hank's Balanced Salt Solution (5 μL, H1045, Solarbio, China). The mixture was immediately dispensed into a 96‐well plate and incubated at 37°C for gelation. After 2 h of incubation, complete medium (100 μL per well) was added. After 72 h, TNT labelling and CLSM imaging were performed.

### 2.16 Identification of Osteocytic TNTs and TUNEL Staining In Vivo

Calvarial bones from 1 to 3‐day‐old C57BL/6J mice were fixed with paraformaldehyde (4%) at 4°C overnight. Samples were then stained with phalloidin at 4°C for 36–48 h, labelled with DiD (100 μM) for 1 week, counterstained with DAPI for 1 h, mounted and imaged by CLSM to visualize TNTs and cargo transport between young osteocytes.

Femora and tibiae from 8 to 12‐week‐old mice were fixed with paraformaldehyde (4%) at 4°C for 24–48 h, and decalcified in EDTA (10%) on a shaker for 3–4 weeks. After gradient cryoprotection in sucrose (10%, 20% and 30%) at 4°C, samples were embedded in OCT (4583, SAKURA, Japan) and 50‐μm‐thick sections were prepared using a cryostat (CM1850, Leica, Germany). Sections were sequentially stained with phalloidin, DiD and DAPI as above, mounted and imaged by CLSM to identify TNTs and assess cargo transport between mature osteocytes. To observe cargo transport between apoptotic and healthy osteocytes, sections were labelled using a TUNEL kit (C1088, Beyotime, China) before DAPI counterstaining and imaging.

### 2.17 Statistical Analysis

All experiments were conducted with at least three independent replicates, and data are presented as the mean ± standard error (SE). Statistical analyses were performed using GraphPad Prism software (version 10.1.0). For comparisons across multiple groups, one‐way analysis of variance (ANOVA) followed by Tukey's post hoc test was applied. Comparisons between two groups were performed using an unpaired two‐tailed Student's *t*‐test. A *p*‐value less than 0.05 was considered statistically significant. *p < 0.05, **p < 0.01, ***p < 0.001; ns, not significant.

## 3 Results

### 3.1 Identification and General Characteristics of TNTs Between Osteocytes

**FIGURE 1:**
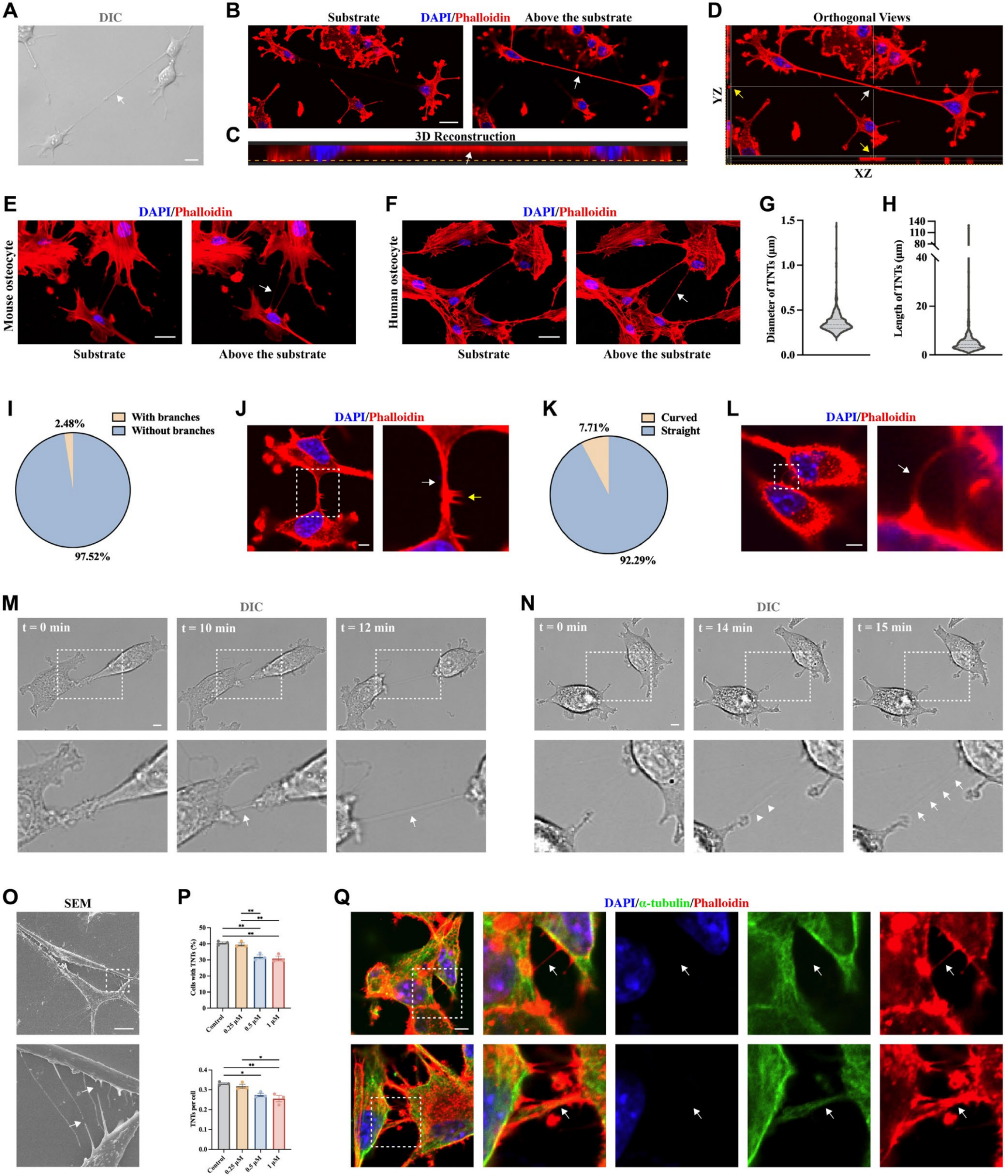
Identification and general characteristics of TNTs between osteocytes. (A) DIC image of a TNT‐like structure (white arrow) connecting MLO‐Y4 cells. Scale bar, 20 μm. (B) Representative CLSM images of a TNT (white arrow). F‐actin, phalloidin (red) and nuclei, DAPI (blue). The left panel shows the dish‐bottom focal plane and the right panel shows a higher focal plane. Scale bar, 20 μm. (C) 3D reconstruction of a TNT (white arrow). The dashed line indicates the dish bottom. (D) Orthogonal view of a TNT (white arrow). Yellow arrows indicate the corresponding XZ and YZ planes. (E, F) Representative CLSM images of a TNT (arrow) between mouse primary osteocytes (E) and between human primary osteocytes (F). Scale bar, 20 μm. (G, H) Quantification of TNT diameter (G) and length (H) in MLO‐Y4 cells (*n* = 363 TNTs). (I) Proportion of TNTs with and without branches (*n* = 363 TNTs). (J) Representative CLSM image of a branched TNT. White arrow indicates the main TNT and yellow arrow indicates the branch structures. Scale bar, 5 μm. (K) Proportion of straight and curved TNTs (*n* = 363 TNTs). (L) Representative CLSM image of a curved TNT (arrow). Scale bar, 5 μm. (M, N) Time‐lapse DIC and live‐cell imaging showing two mechanisms of TNT formation: Cell dislodgement (M) and protrusion elongation (N). Arrowheads indicate a filopodia‐like protrusion that did not connect to the target cell and arrows indicate the TNT that connected to the target cell. Scale bar, 5 μm. (O) SEM image of TNT formation or breakage. Scale bar, 10 μm. (P) Effects of cytochalasin B on TNT formation (*n* = 3). (Q) CLSM images of TNTs lacking α‐tubulin (upper panel) or containing α‐tubulin (lower panel). Scale bar, 5 μm. Data are presented as mean ± SE. Statistical significance was assessed using ANOVA with Tukey's post hoc test. *p < 0.05, **p < 0.01, ***p < 0.001.

First, we systematically identified TNTs in the MLO‐Y4 osteocyte‐like cells. Under DIC microscopy, thin tubular connections were observed between adjacent cells (Figure 1A). CLSM with Z‐stack imaging revealed that these F‐actin–positive structures were absent at the dish‐bottom focal plane but became clearly visible above the substrate (Figure 1B). Three‐dimensional (3D) reconstruction and orthogonal views further confirmed that they did not contact the substrate (Figure 1C,D). These features meet the established criteria for TNTs. Similarly, TNTs were also observed between mouse and human primary osteocytes (Figures 1E,F and S1).

Given their accessibility and widespread use in osteocyte research [3, 38, 39, 40, 41], MLO‐Y4 cells were used as the principal model for subsequent analyses. TNT diameters ranged from 0.21 to 1.43 μm, while their lengths were highly heterogeneous, ranging from 1.12 to 128.95 μm (Figure 1G,H). Most TNTs lacked branching structures (97.52%) and were straight (92.29%) (Figure 1I–L). Time‐lapse imaging revealed two distinct modes of formation: cell dislodgement (cells separate while remaining connected) (Figure 1M) and protrusion elongation (a filopodia‐like extension contacts a neighbouring cell) (Figure 1N). Additionally, SEM captured TNTs in apparent stages of formation or rupture (Figure 1O), supporting their dynamic nature. Because F‐actin is a core component of TNTs, we further investigated whether actin polymerization is required for their formation. Treatment with cytochalasin B, an inhibitor of actin polymerization, revealed that 0.5 and 1 μM markedly reduced both the percentage of TNT‐positive cells and the average number of TNTs per cell, without affecting cell viability (Figures 1P and S2A). Thus, osteocytic TNT formation is partially dependent on actin polymerization. Immunofluorescence staining showed that most TNTs were α‐tubulin‐negative and relatively thin, while a subset of thick TNTs showed positive α‐tubulin staining (Figure 1Q), suggesting compositional differences among TNTs of varying diameters. In summary, we identified bona fide TNTs between osteocytes and defined their morphology, formation dynamics and cytoskeletal composition.

### 3.2 Specific Features of Osteocytic TNTs

**FIGURE 2:**
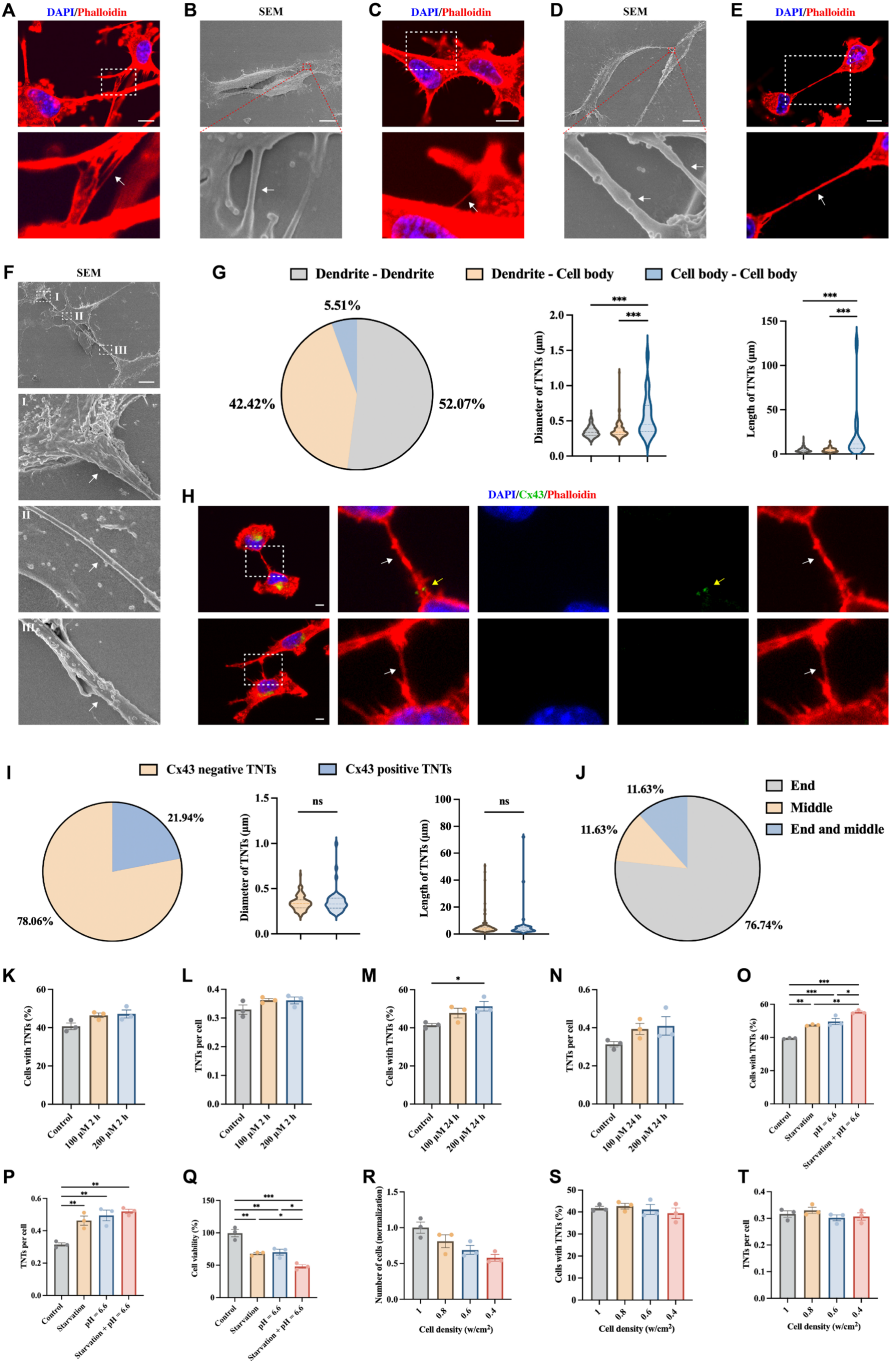
Osteocyte‐specific characteristics of TNTs. (A) CLSM image of TNTs (arrows) connecting the dendrites of MLO‐Y4 cells. F‐actin, phalloidin (red); nuclei, DAPI (blue). (B) SEM image of TNTs connecting dendrites. (C, D) CLSM (C) and SEM (D) images of TNTs connecting a dendrite and a cell body. (E, F) CLSM (E) and SEM (F) images of TNTs connecting cell bodies. (G) Quantification of the proportion (left), diameter (middle) and length (right) of TNTs by connection site (*n* = 363 TNTs). (H) CLSM images of TNTs containing (upper) and lacking Cx43 (lower). White arrows indicate TNTs and yellow arrows indicate Cx43. (I) Quantification of the proportion (left), diameter (middle) and length (right) of Cx43‐negative and Cx43‐positive TNTs (*n* = 196 TNTs). (J) Distribution of Cx43 along TNTs (*n* = 43 TNTs). (K, L) Effects of 100 and 200 μM H_2_O_2_ treatment for 2 h on the percentage of cells with TNTs (K) and the average number of TNTs per cell (L) (*n* = 3). (M, N) Effects of 100 and 200 μM H_2_O_2_ treatment for 24 h on the percentage of cells with TNTs (M) and the average number of TNTs per cell (N) (*n* = 3). (O–Q) Effects of serum starvation and/or acidic medium on the percentage of cells with TNTs (O), the average number of TNTs per cell (P) and cell viability (Q) (*n* = 3). (R–T) Cell count (R), percentage of cells with TNTs (S) and average number of TNTs per cell (T) at different seeding densities (*n* = 3). All scale bars, 10 μm. Data are presented as mean ± SE. Statistical significance was assessed using one‐way ANOVA with Tukey's post hoc test for multiple group comparisons, and an unpaired two‐tailed Student's t‐test for two‐group comparisons. *p < 0.05, **p < 0.01, ***p < 0.001; ns, not significant.

Since osteocytes possess a unique morphology characterized by a central cell body and multiple dendrites, we next investigated whether osteocytic TNTs show site‐specific connectivity. CLSM and SEM revealed that TNTs could form between dendrites of different cells (Figure 2A,B), between dendrites and cell bodies (Figure 2C,D) and between two cell bodies (Figure 2E,F). SEM further showed that all TNT ends exhibited continuous plasma membrane fusion (Figure 2B,D,F). Quantification demonstrated that the majority of TNTs were formed between dendrites or between dendrites and cell bodies, while connections between cell bodies accounted for only 5.51% (Figure 2G). Notably, TNTs connecting cell bodies were thicker and longer than dendrite‐associated TNTs (Figure 2G). Thus, TNTs connecting different cellular sites vary in both diameter and length, which may reflect distinct functional roles in intercellular communication. Connexin 43 (Cx43), a gap junction protein enriched in osteocyte dendrites, mediates canonical osteocytic communication [42, 43]. We then assessed Cx43 expression on TNTs. Approximately 21.94% of TNTs exhibited Cx43‐positive signals, with no significant differences in either diameter or length between Cx43‐positive and Cx43‐negative TNTs (Figure 2H,I). Interestingly, Cx43 signals were predominantly localized at the terminal ends of TNTs (Figure 2J).

Although TNTs can spontaneously form, osteocytes are frequently exposed to stressful microenvironments in vivo, which have been reported to promote TNT formation [43]. We therefore examined osteocytic TNT formation under stress conditions. Under oxidative stress, treatment with 200 μM H_2_O_2_ for 24 h modestly increased the percentage of cells with TNTs, with no viability loss (Figures 2K–N and S2B,C). Both serum starvation and acidic culture conditions significantly increased the percentage of cells with TNTs and the average number of TNTs per cell; when combined, TNT formation was further enhanced (Figure 2O,P). These conditions also reduced cell viability (Figure 2Q). To rule out a density effect, we varied seeding density. TNT formation remained stable across different densities, indicating that the observed changes were not density driven (Figure 2R–T). Together, these data indicate that stress promotes osteocytic TNT formation, suggesting an adaptive response to adverse microenvironments.

### 3.3 TNT‐Mediated Transfer of Membrane‐Bound Cargo Between Osteocytes

**FIGURE 3:**
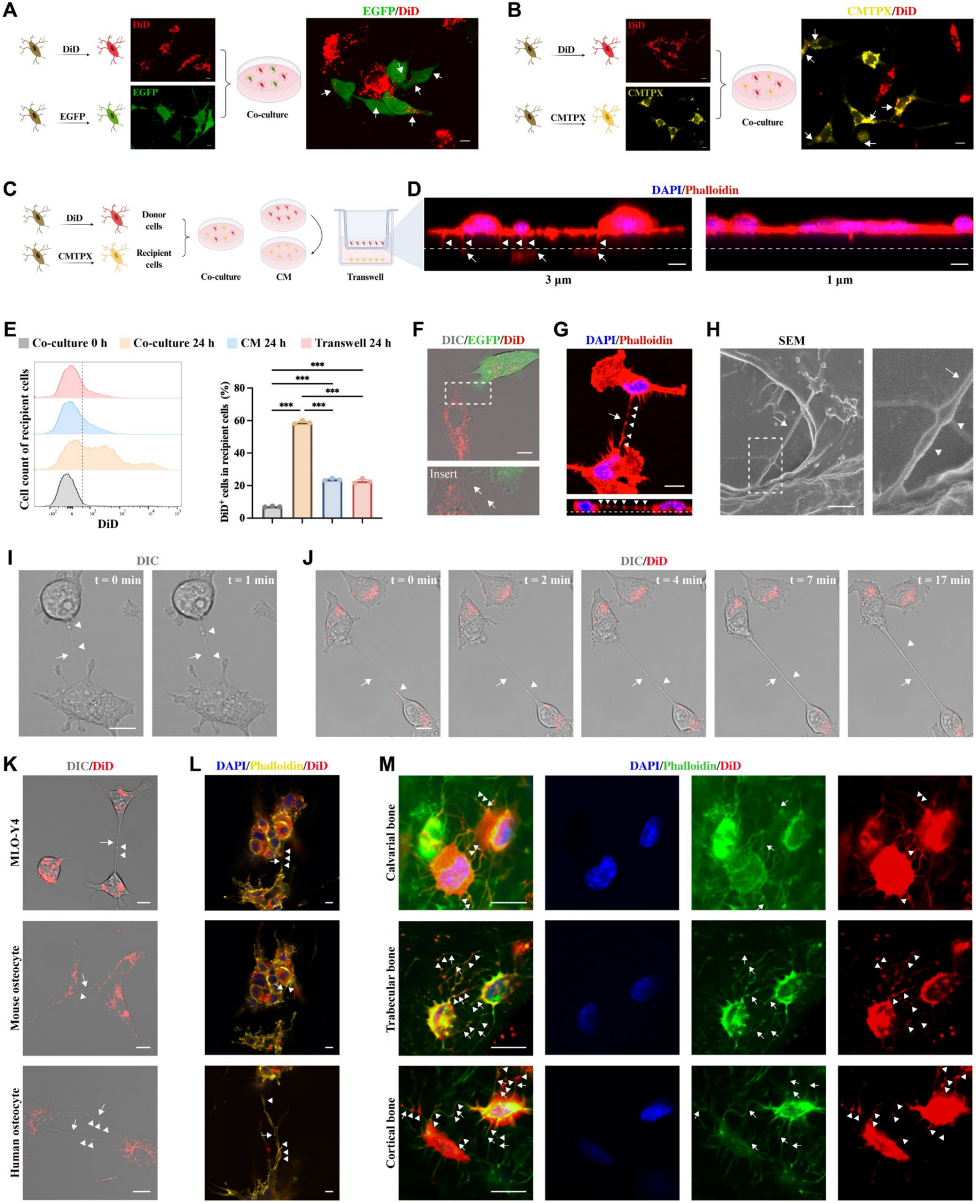
Osteocytic TNTs mediate intercellular transfer of membrane‐bound cargo. (A, B) Schematic diagram and CLSM images of co‐cultured DiD‐labelled donor cells (red) with EGFP‐labelled (A, green) or CMTPX‐labelled (B, yellow) recipient cells. Arrows indicate DiD^+^ signals within recipient cells. Scale bar, 10 μm. (C) Schematic diagram of direct, CM and Transwell co‐culture systems. (D) CLSM images of the Transwell membrane after co‐culture using different pore sizes. The dashed line indicates the lower membrane surface, arrowheads indicate TNTs extending into membrane pores, and arrows indicate TNTs traversing the lower membrane layer. Scale bar, 10 μm. (E) Flow cytometry histograms of DiD fluorescence in CMTPX‐labelled recipient cells and quantification of the percentage of DiD^+^ cells in recipient cells after 0 and 24 h of co‐culture (*n* = 3). (F) CLSM image of a donor cell and an EGFP‐labelled recipient cell connected via TNTs (arrows), with DiD^+^ signals detected in the recipient cell. Scale bar, 10 μm. (G) CLSM image showing bead‐like structures (arrowheads) within a TNT (arrow). Upper, higher focal plane; lower, 3D reconstruction. The dashed line indicates the dish bottom. Scale bar, 10 μm. (H) SEM image showing bead‐like structures (arrowheads) within a TNT (arrow). Scale bar, 1 μm. (I) Live‐cell imaging showing dynamic movement of bead‐like structures (arrowheads) within a TNT (arrow). Scale bar, 10 μm. (J) Time‐lapse imaging of a DiD‐labelled membrane‐bound cargo (arrowhead) moving continuously along a TNT (arrow). Scale bar, 10 μm. (K) Representative CLSM images of DiD‐labelled cargo (arrowheads) transported via TNTs (arrows) in MLO‐Y4 cells (upper), mouse primary osteocytes (middle) and human primary osteocytes (lower panel). Scale bar, 10 μm. (L) CLSM images showing TNTs (arrows) and DiD‐labelled cargo (arrowheads) transported between MLO‐Y4 cells in 3D culture. The upper, middle and lower panels represent XY optical sections at different *Z*‐axis levels. Scale bar, 10 μm. (M) CLSM images showing TNTs (arrows) and DiD‐labelled cargo (arrowheads) transported in vivo in the calvarial bone of neonatal mice (upper panel), as well as trabecular bone (middle panel) and cortical bone (lower panel) of adult mice. Scale bar, 10 μm. Data are presented as mean ± SE. Statistical significance was assessed using one‐way ANOVA with Tukey's post hoc test. ***p < 0.001.

To assess TNT‐mediated cargo transfer, we used DiD (a lipophilic membrane tracer that labels membrane‐bound structures) to label donor osteocytes and co‐cultured them with EGFP‐transfected or CMTPX‐labelled recipient osteocytes (Figure 3A,B) [36]. After 24 h, most recipient cells exhibited DiD^+^ fluorescence, while donor cells showed almost no EGFP or CMTPX signals (Figure 3A,B), indicating extensive transfer of membrane‐bound structures but not cytoplasmic content between osteocytes. To distinguish direct from indirect transfer, we compared direct co‐culture with two indirect co‐culture systems, including conditioned medium (CM) and Transwell co‐culture (Figure 3C). In the Transwell system, 3‐μm pore inserts allowed TNT‐like structures to traverse the membrane, whereas 1‐μm pore inserts effectively blocked such direct connections; therefore, 1‐μm pore inserts were used in subsequent Transwell co‐culture experiments (Figure 3D). Flow cytometry showed that direct co‐culture markedly elevated the proportion of DiD^+^ cells in the recipient cells to 58.77%, significantly higher than both the 0 h and indirect co‐culture groups (Figure 3E). Thus, transfer of DiD^+^ cargo predominantly occurs via direct cell‐to‐cell communication.

We next tested whether cargo transfer is mediated specifically via TNTs. In direct co‐cultures, TNTs connected DiD‐labelled donor cells and EGFP‐labelled recipient cells, with DiD signals clearly visible in the recipient cytoplasm (Figure 3F), suggesting TNT‐mediated transfer. Bead‐like structures, presumed to represent membrane‐bound cargo in transit, were visualized within TNTs by CLSM and SEM (Figure 3G,H). Live‐cell imaging further captured their dynamic, directional movement along TNTs (Figure 3I). Time‐lapse imaging of DiD‐labelled osteocytes revealed continuous transport of membrane‐bound cargo along TNTs (Figure 3J), and in some cases, complete transport of an individual DiD^+^ punctum between cells was recorded (Figure 3K, upper panel, Figure S3, Movie S1). In contrast, complete transport of DiD^+^ cargo along dendrites was not observed (data not shown), indicating that TNTs are the primary conduits mediating the transfer of membrane‐bound cargo between osteocytes. Similar TNT‐mediated cargo transport was observed in mouse and human primary osteocytes (Figure 3K, middle and lower panels).

We further evaluated TNT‐mediated transfer beyond 2D culture. TNTs were observed within 3D collagen matrices, with visible transport of DiD^+^ cargo (Figure 3L). Furthermore, imaging of calvarial tissue from postnatal Day 2 mice revealed DiD^+^ cargo within TNT‐like structures that connect young osteocytes (Figure 3M, upper panel). Similarly, in both trabecular and cortical bone of adult mouse femora, TNTs and associated cargo transport were consistently detected between mature osteocytes (Figure 3M, middle and lower panels). Taken together, these findings, spanning in vitro cell lines, primary osteocytes, 3D culture and in vivo bone tissue, demonstrate that osteocytic TNTs mediate active intercellular transfer of membrane‐bound cargo.

### 3.4 Osteocytic TNT‐Mediated Transfer of Membrane‐Bound Cargo Rescues Cells From Apoptosis

**FIGURE 4:**
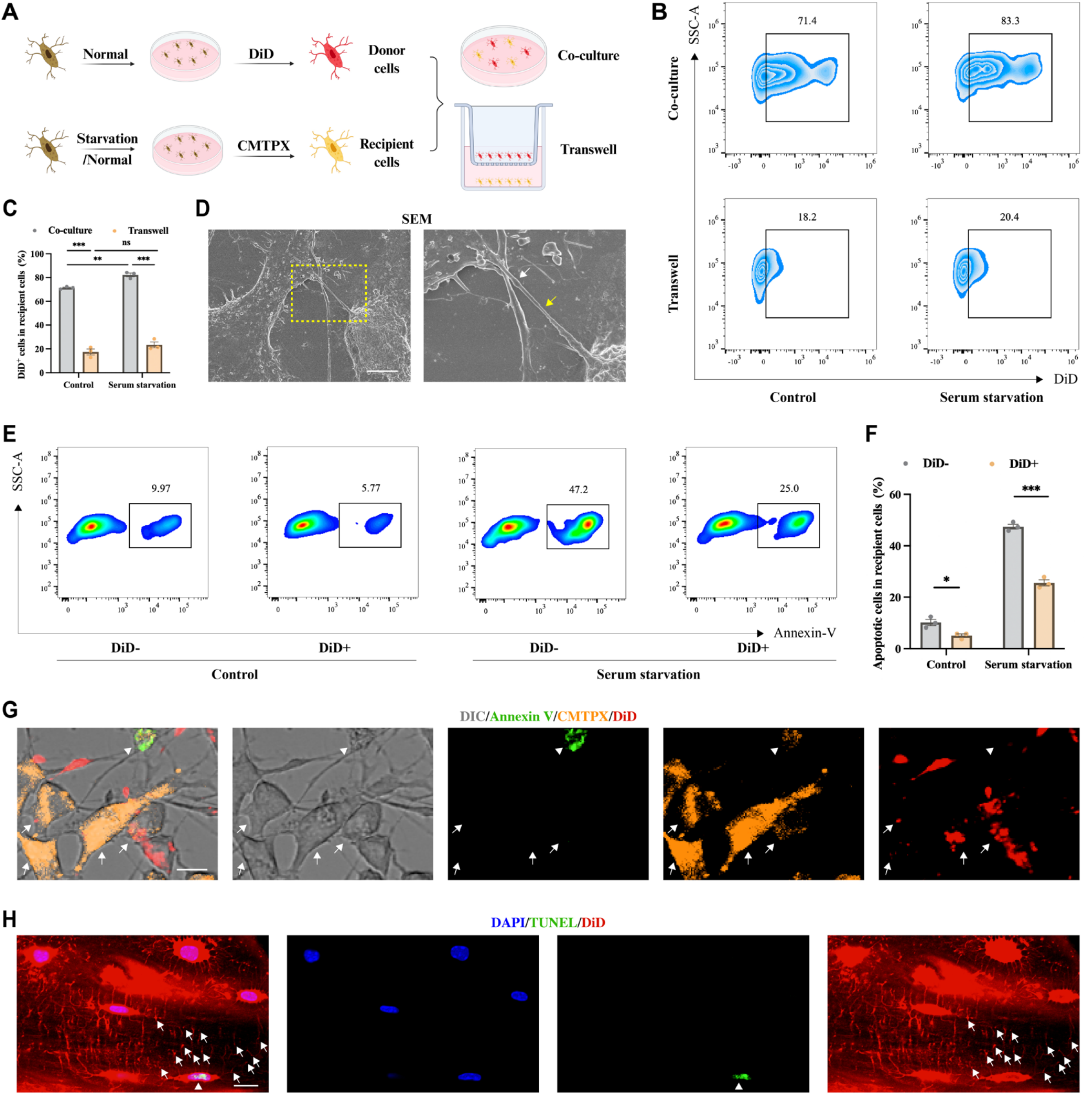
Osteocytes transfer membrane‐bound cargo via TNTs to prevent apoptosis. (A–C) Schematic diagram (A), flow cytometry plots (B), and quantification (C) of the percentage of DiD^+^ cells in recipient cells in direct or Transwell co‐culture of DiD‐labelled donor cells (red) with CMTPX‐labelled recipient cells (yellow) under normal or serum‐starved conditions (*n* = 3). (D) SEM image of TNT‐mediated cargo transport between a normal osteocyte and a dying osteocyte. Yellow arrow indicates a TNT and white arrow indicates transported cargo. Scale bar, 5 μm. (E, F) Flow cytometry plots of Annexin V signals in recipient cells (E) and quantification of the percentage of Annexin V‐positive cells in recipient cells (F) from co‐cultures of normally cultured donor cells with serum‐starved or normally cultured recipient cells (*n* = 3). (G) Representative CLSM image of direct co‐cultures of normally cultured donor cells with serum‐starved recipient cells. Arrows indicate DiD^+^ recipient cells; arrowheads indicate DiD^−^ recipient cells. Scale bar, 10 μm. (H) Representative CLSM image of DiD^+^ cargo transport (arrows) between a healthy osteocyte and a TUNEL‐positive apoptotic osteocyte (arrowhead) in mouse femoral cortical bone in vivo. Scale bar, 10 μm. Data are presented as mean ± SE. Statistical significance was assessed using an unpaired two‐tailed Student's *t*‐test. *p < 0.05, **p < 0.01, ***p < 0.001; ns, not significant.

Our previous findings demonstrated that nutrient deprivation increases TNT formation (Figure 2O,P). To test whether stress‐induced TNT formation promotes cargo transfer, we co‐cultured serum‐starved recipient osteocytes with DiD‐labelled healthy donors (Figure 4A). Flow cytometry revealed that serum starvation significantly increased the percentage of DiD^+^ cells in recipient cells in direct co‐culture but not in Transwell conditions (Figure 4B,C), suggesting that stressed osteocytes receive a markedly greater number of DiD^+^ cargo from healthy donor cells via TNTs. Consistent with this, cargo in transit was directly visualized within TNTs connecting healthy and dying osteocytes (Figure 4D).

To assess the functional consequence of this transfer, we quantified apoptosis in recipient cells. In serum‐starved co‐cultures, DiD^+^ recipient cells exhibited significantly lower apoptosis (25.60%) than DiD^−^ recipients (47.43%) (Figure 4E,F), demonstrating that TNT‐transferred DiD^+^ cargo protects stressed osteocytes from apoptosis. Notably, even under normal culture conditions, DiD^+^ recipient cells exhibited a modestly lower basal apoptosis rate than DiD^−^ recipient cells (Figure 4E,F), suggesting that TNT‐mediated DiD^+^ cargo transfer may also contribute to homeostatic maintenance. CLSM corroborated this pattern: DiD^+^ recipient cells were predominantly Annexin V‐negative, whereas DiD^−^ recipient cells were essentially Annexin V‐positive (Figure 4G). Similar phenomena were observed in vivo: extensive DiD^+^ cargo transport was observed between healthy osteocytes and TUNEL‐positive osteocytes in mouse bone tissue (Figure 4H). In summary, stress promotes TNT‐mediated transfer of membrane‐bound cargo from healthy to compromised osteocytes, thereby reducing apoptosis and supporting osteocyte survival.

### 3.5 The Membrane‐Bound Cargo Transferred by Osteocytes via TNTs Consists Primarily of Lysosomes

**FIGURE 5:**
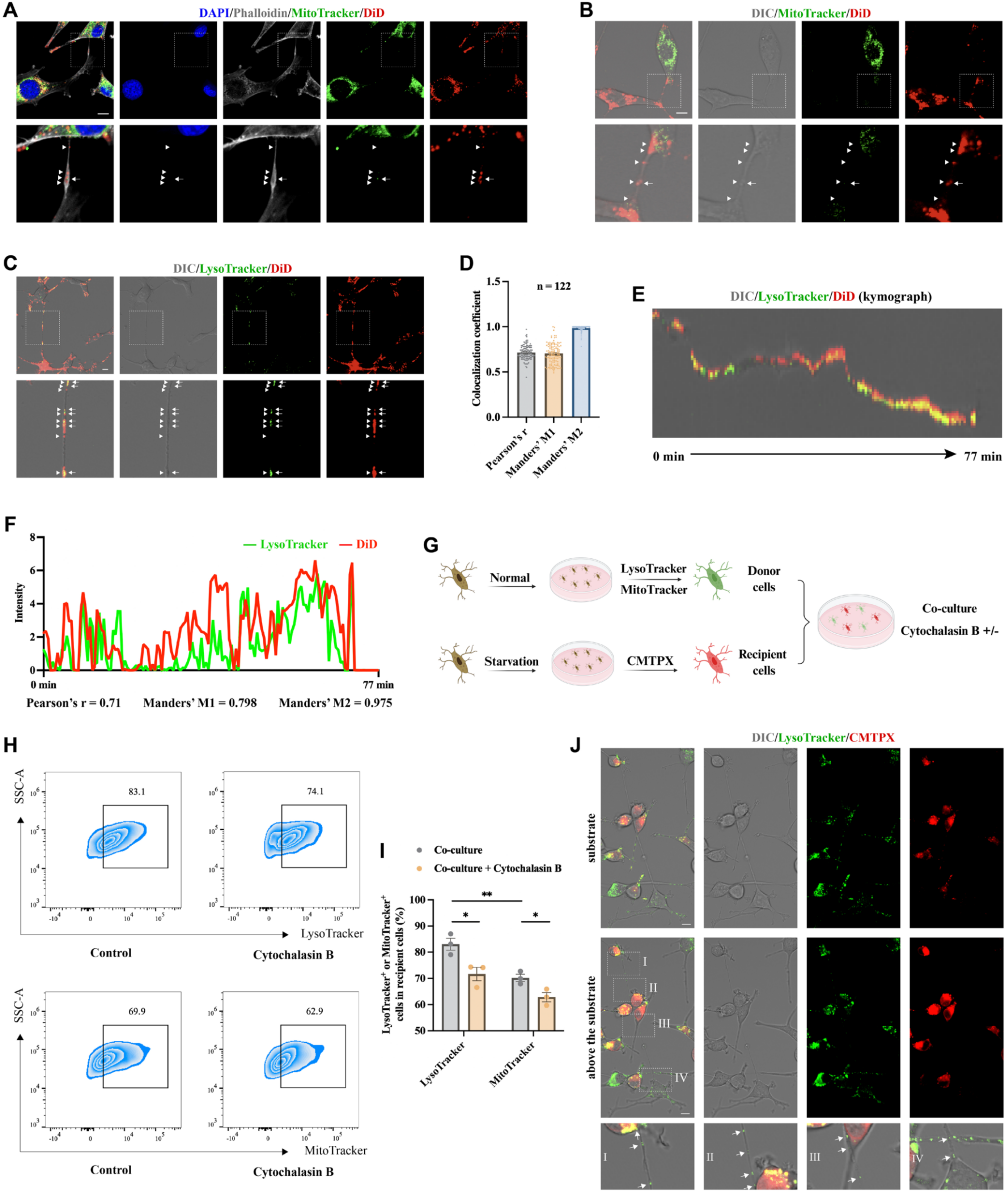
Osteocytes transfer lysosomes via TNTs. (A, B) Representative CLSM image (A) and live‐cell imaging (B) of intercellular transport of MitoTracker‐labelled mitochondria (arrow) and DiD‐labelled membrane‐bound cargo (arrowheads) via TNTs. Scale bar, 10 μm. (C) Representative live‐cell imaging of LysoTracker‐labelled lysosomes (arrows) and DiD‐labelled cargo (arrowheads) transported via TNTs. Scale bar, 10 μm. (D) Quantitative analysis of colocalization between DiD and LysoTracker signals within TNTs, shown as Pearson's r, Manders' M1 (fraction of total DiD intensity overlapping with LysoTracker), and Manders' M2 (fraction of total LysoTracker intensity overlapping with DiD) coefficients (*n* = 122 TNTs). (E) Representative kymograph of the middle region of a TNT, showing the dynamic trajectories of DiD and LysoTracker signals passing through this region during live‐cell imaging. (F) Corresponding signal intensity profiles of DiD and LysoTracker and the colocalization analysis shown in (E). (G–I) Schematic diagram (G), flow cytometry plots (H) and quantification (I) of the percentage of LysoTracker^+^ or MitoTracker^+^ cells in recipient cells in co‐cultures of normally cultured donor cells (green) labelled with LysoTracker or MitoTracker and CMTPX‐labelled recipient cells (red) following serum starvation, with or without cytochalasin B treatment (*n* = 3). (J) Representative CLSM images of lysosomes (arrows) transported within TNTs in co‐culture. Scale bar, 10 μm. Data are presented as mean ± SE. Statistical significance was assessed using an unpaired two‐tailed Student's *t*‐test. *p < 0.05, **p < 0.01.

Because DiD is a lipophilic dye that labels membrane‐bound structures, and prior studies have reported that mitochondria and lysosomes are the major membrane‐bound organelles transferred through TNTs [44, 45], we investigated mitochondrial and lysosomal transfer. In osteocytes co‐labelled with MitoTracker and DiD, DiD^+^ signals were frequently detected along TNTs, whereas mitochondrial transport events were less frequent (Figure 5A,B). We then co‐labelled osteocytes with LysoTracker and DiD, revealing that abundant lysosomes were transported along TNTs and colocalized with DiD^+^ cargo (Figure 5C). Further colocalization analysis of 122 TNTs showed strong correlation and a high degree of overlap between DiD and LysoTracker signals (Figure 5D). In addition, kymograph analysis of the middle region of a TNT revealed highly concordant DiD and LysoTracker trajectories through this region, indicating clear colocalization between the two signals (Pearson's *r* = 0.71, Manders' M1 = 0.798 and Manders' M2 = 0.975) (Figure 5E,F). Lipophilic dyes with long octadecyl chains, such as DiD, are known to preferentially accumulate in lysosomal compartments following cellular uptake [46]. Together, these findings suggest that the DiD^+^ cargo transferred via TNTs consists predominantly of lysosomes.

To further quantitatively compare the transfer efficiency of lysosomes and mitochondria, donor cells labelled with LysoTracker or MitoTracker were co‐cultured with CMTPX‐labelled recipient osteocytes that had been serum‐starved, with or without cytochalasin B (Figure 5G). Flow cytometry analysis revealed that 83.03% of recipient cells acquired lysosomes, whereas 71.63% acquired mitochondria (Figure 5H,I), indicating that osteocytes preferentially transfer lysosomes over mitochondria under stress conditions. Cytochalasin B significantly decreased the proportion of recipient cells that received lysosomes to 70.17%, confirming that lysosome transfer is largely TNT‐mediated. Similarly, cytochalasin B also reduced mitochondrial transfer (Figure 5H,I), indicating that although mitochondrial transfer occurs less frequently, it is still facilitated by TNTs. Furthermore, CLSM vividly demonstrated the dynamic transport of large amounts of lysosomes along TNTs between osteocytes (Figure 5J). Collectively, the above results demonstrate that the membrane‐bound cargo transferred through TNTs between osteocytes consists primarily of lysosomes and that this form of intercellular communication occurs extensively within osteocyte networks.

### 3.6 Osteocytic TNT‐Mediated Lysosome Transfer Prevents Apoptosis Through Transcellular Autophagy

**FIGURE 6:**
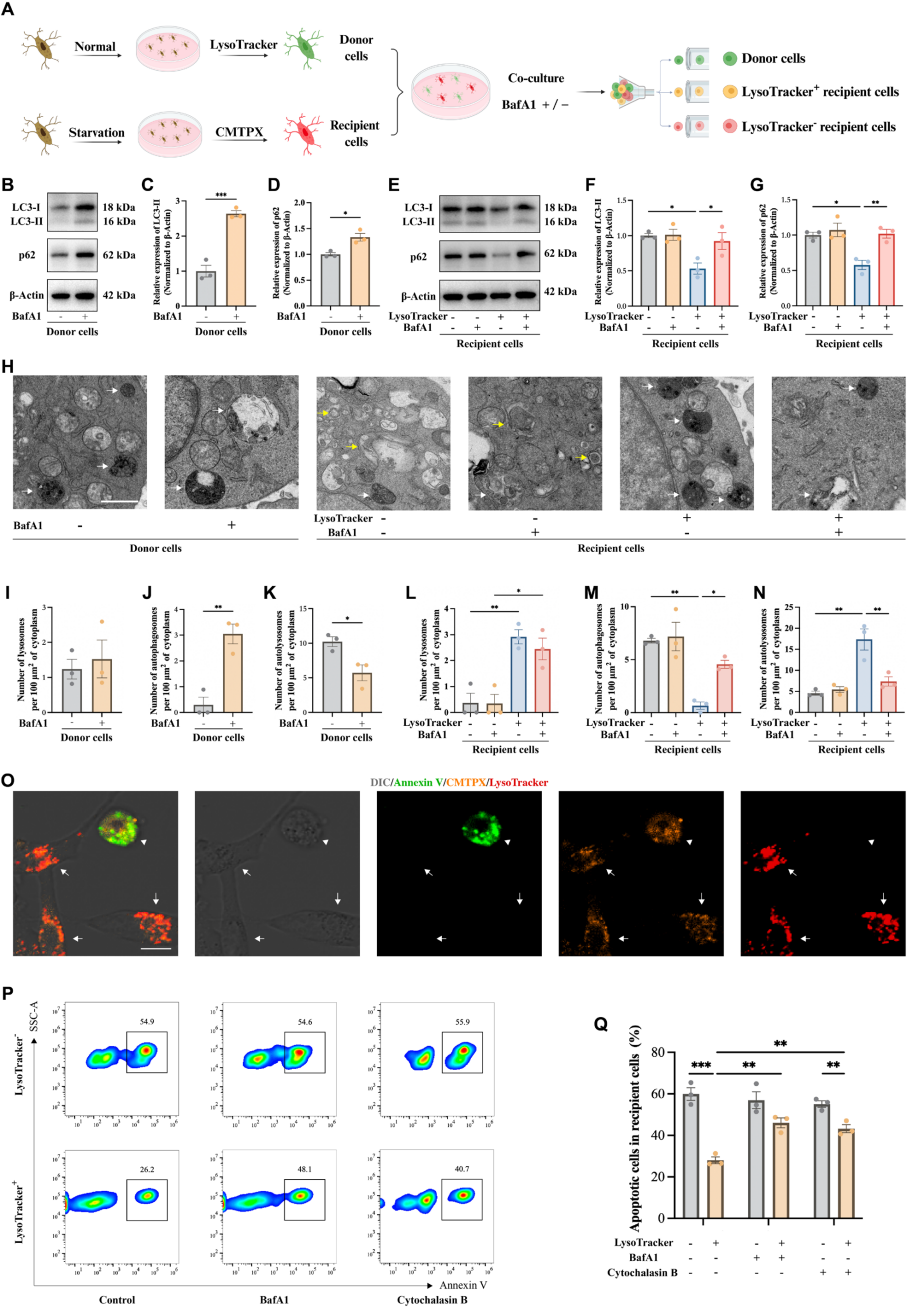
Osteocytes transfer lysosomes via TNTs to promote autophagy and prevent apoptosis. (A) Schematic diagram of the co‐culture and FACS strategy using LysoTracker‐labelled donor cells (green) and CMTPX‐labelled recipient cells (red) subjected to serum starvation, with or without BafA1 treatment. FACS gates: CMTPX^−^/LysoTracker^+^ cells (donor cells), CMTPX^+^/LysoTracker^+^ cells (LysoTracker^+^ recipient cells) and CMTPX^+^/LysoTracker^−^ cells (LysoTracker^−^ recipient cells). (B) Western blot detection of autophagy‐related proteins in donor cells after FACS. (C, D) Semi‐quantification of LC3‐II (C) and p62 (D) protein levels in donor cells (*n* = 3). (E) Western blot detection of autophagy‐related proteins in recipient cells after FACS. (F, G) Semi‐quantification of LC3‐II (F) and p62 (G) protein levels in recipient cells (*n* = 3). (H) Representative TEM images of autophagy‐related structures after FACS. White arrows indicate autolysosomes and yellow arrows indicate autophagosomes. Scale bar, 1 μm. (I–K) Quantification of lysosomes (I), autophagosomes (J) and autolysosomes (K) per 100 μm^2^ of cytoplasm in donor cells (*n* = 3). (L–N) Quantification of lysosomes (L), autophagosomes (M) and autolysosomes (N) per 100 μm^2^ of cytoplasm in recipient cells (*n* = 3). (O) Representative CLSM image of Annexin V staining in co‐cultures of LysoTracker‐labelled donor cells with CMTPX‐labelled recipient cells under serum starvation. Arrows indicate LysoTracker^+^ recipient cells; arrowhead indicates a LysoTracker^−^ recipient cell. Scale bar, 10 μm. (P, Q) Flow cytometry plots of Annexin V signals in recipient cells (P) and quantification of the percentage of Annexin V‐positive cells in recipient cells (Q) in co‐cultures of normally cultured donor cells and recipient cells under serum‐starvation, with or without BafA1 or cytochalasin B treatment (*n* = 3). Data are presented as mean ± SE. Statistical significance was assessed using one‐way ANOVA with Tukey's post hoc test for multiple group comparisons and an unpaired two‐tailed Student's t‐test for two‐group comparisons. *p < 0.05, **p < 0.01, ***p < 0.001; ns, not significant.

Since lysosomes are essential for autophagic degradation [47], we next investigated whether TNT‐transferred lysosomes can functionally participate in autophagy in recipient cells (Figure 6A). In FACS‐sorted populations, donor cells exhibited low basal LC3‐II and p62 levels, which markedly increased upon BafA1 treatment (Figure 6B–D), confirming intact basal autophagic flux. In contrast, LysoTracker^−^ recipient cells showed high LC3‐II and p62 levels, which were not further increased by BafA1 (Figure 6E–G), indicating a blockade of autophagic flux. Strikingly, LysoTracker^+^ recipient cells exhibited significantly reduced LC3‐II and p62 levels, whereas BafA1 induced robust accumulation of both proteins (Figure 6E–G). TEM of sorted cells supported these findings (Figure 6H–N). Donor osteocytes showed predominantly autolysosomes, whereas BafA1 caused autophagosome accumulation, consistent with intact basal autophagic flux (Figure 6H–K). In contrast, LysoTracker^−^ recipient cells contained scant lysosomes but abundant autophagosomes, with few autolysosomes, a state unaffected by BafA1 (Figure 6H,L–N). However, LysoTracker^+^ recipient cells showed enhanced lysosomes (Figure 6H,L), reduced autophagosomes and increased autolysosomes, reflecting reactivation of autophagic flux. Upon BafA1 treatment, autophagosomes again accumulated while autolysosomes decreased (Figure 6H,L–N). These results identify starvation‐induced lysosome deficiency as a key cause of autophagy blockade in stressed osteocytes. TNT‐mediated lysosome sharing from healthy donor cells restores autophagosome–lysosome fusion and autophagic degradation and enhances autophagic activity. This process establishes a transcellular autophagy pathway, in which exogenous lysosomes mediate autophagic clearance.

Based on our previous observations that osteocytic transfer of membrane‐bound cargo rescues recipient cells from apoptosis, and that this cargo is predominantly lysosomes, we hypothesised that TNT‐mediated lysosome sharing suppresses apoptosis by restoring autophagic flux in stressed cells. CLSM imaging revealed numerous lysosomes being transported within TNTs between healthy and serum‐starved osteocytes (Figure S4). Annexin V staining showed strong apoptotic signals in LysoTracker^−^ recipient cells, while the LysoTracker^+^ recipient cells were Annexin V‐negative (Figure 6O). Flow cytometry confirmed that LysoTracker^−^ recipient cells exhibited an apoptosis rate of 59.93%, whereas the LysoTracker^+^ recipient cells showed a significantly reduced rate of 28.00% (Figure 6P,Q). Thus, lysosome transfer effectively alleviated stress‐induced apoptosis. Significantly, BafA1 increased apoptosis in LysoTracker^+^ recipient cells to 46.03%, proving that the cytoprotective effect by lysosome transfer depends on restoration of autophagic flux. Furthermore, inhibition of TNT formation with cytochalasin B increased apoptosis to 43.23% (Figure 6P,Q), indicating that disruption of TNTs is sufficient to impair lysosome transfer and abolish its subsequent anti‐apoptotic effect. Collectively, these results demonstrate that the transcellular autophagy pathway significantly suppresses apoptosis and promotes survival by restoring autophagic flux.

To verify whether this rescue effect is organelle‐specific, we investigated the impacts of mitochondrial transfer (Figure S5A). Donor cells displayed low basal LC3‐II and p62 levels, which accumulated upon BafA1 addition (Figure S5B–D), indicating intact autophagic flux. However, in recipient cells, both MitoTracker^+^ and MitoTracker^−^ recipient cells maintained high LC3‐II and p62 levels, and BafA1 produced no further increase (Figure S5E–G). Correspondingly, both groups exhibited similarly high apoptosis rates, with no significant difference (Figure S5H,I). Thus, although mitochondria can be transferred via TNTs, they fail to restore autophagic flux or reduce apoptosis. Overall, our study establishes TNT‐mediated lysosome transfer as a specific and indispensable mechanism of transcellular autophagy that maintains osteocyte survival by reinstating autophagic flux, whereas mitochondrial transfer lacks this protective function.

## 4 Discussion

**FIGURE 7:**
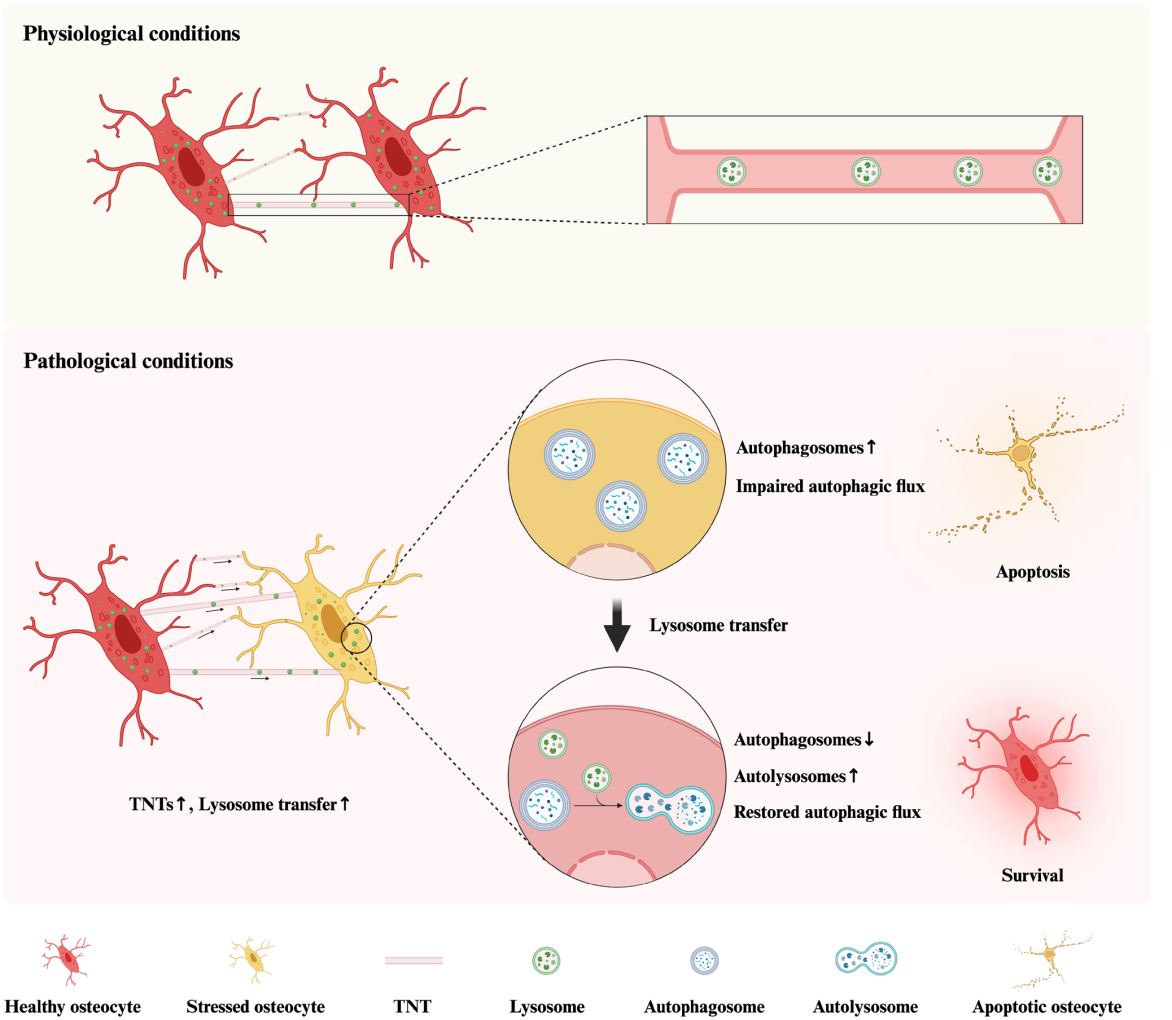
Schematic diagram showing that TNT‐mediated lysosome sharing promotes osteocyte survival via transcellular autophagy. Created in BioRender.

Osteocyte viability is central to bone homeostasis and osteocyte apoptosis contributes to diverse bone diseases. However, how osteocytes maintain long‐term viability within the nutrient‐limited microenvironment remains unclear [8]. Here, we provide definitive evidence for bona fide TNTs between cultured osteocytes and show that they mediate transcellular autophagy via intercellular lysosomal sharing. This TNT‐mediated lysosome transfer occurs under physiological conditions and is markedly enhanced by pathological stress, including nutrient deprivation. Donor‐derived lysosomes fuse with accumulated autophagosomes in stressed recipient cells, restoring autophagic flux and suppressing apoptosis (Figure 7). Together, these findings establish TNT‐dependent lysosome sharing as a core adaptive survival strategy in osteocytes under stress. This mechanism provides a novel framework for bone homeostasis and suggests therapeutic opportunities for disorders driven by osteocyte apoptosis and impaired bone remodelling.

In recent years, TNTs have emerged as a novel form of intercellular communication capable of mediating the transfer of cellular components in many cell types [17]. However, whether bona fide TNTs exist between osteocytes has remained unclear. Gao et al. [9] proposed that osteocyte dendrites could represent TNT‐like protrusions [48]. Nevertheless, dendrites in cultured osteocytes typically adhere to the substrate, whereas TNTs are defined as suspended structures connecting two cells. Thus, dendrites do not strictly meet the morphological criteria for TNTs. In this study, we used F‐actin staining combined with Z‐stack CLSM and 3D reconstruction to systematically identify bona fide TNT structures in cultured osteocytes (Figure 1B–F). Further, we used DIC time‐lapse imaging to visualize TNT formation and cargo transport between osteocytes in real time while minimizing phototoxicity and photobleaching, a strategy widely used to detect TNTs in living cells [36, 49].

To date, direct in vivo evidence for TNTs remains limited [50]. Major obstacles include the lack of specific markers to distinguish TNTs from morphologically similar structures, the extremely thin architecture of TNTs and physical obstruction by the extracellular matrix and surrounding tissues, which hampers visualization [29, 45, 51]. These challenges are particularly pronounced in osteocyte research, where investigators must contend with the optical opacity of mineralized bone and its inherent physical shielding at the tissue level [52]. Nevertheless, our data offer compelling insight into the potential presence of TNTs within bone. Bone canaliculi can reach a maximum diameter of nearly 0.5 μm [53], whereas TNTs between MLO‐Y4 osteocytes exhibited a mean diameter of ~0.366 μm, with the thinnest measuring 0.21 μm (Figure 1G). Thus, a significant proportion (92.56%) of TNTs may be sufficiently small in size to fit within the canalicular network. Furthermore, the recent identification of “canalicular junctions” strengthens this possibility [54], especially for dendrite‐dendrite TNTs, which represent the predominant subtype observed in vitro. It should be noted, however, that TNT dimensions measured in vitro may not fully reflect those of TNTs formed within the spatially constrained 3D environment of the mineralized bone matrix. Currently, a generally accepted criterion for identifying TNTs in vivo is the visualization of tubular F‐actin–positive structures that transport cargo between connected cells [29, 55]. In accordance with this criterion, our imaging analyses of bone tissue revealed TNT‐like structures that not only connected neighbouring osteocytes but also contained membrane‐bound cargo in transit (Figure 3M). While previous in vivo studies have commonly referred to similar structures as ‘dendrites’ [56], the presence of transferred cargo supports the interpretation that these structures may represent TNTs according to currently accepted in vivo functional and morphological criteria. Nevertheless, conclusively distinguishing TNTs from traditional dendrites within the dense and optically complex bone matrix requires further technological advances. Future work should establish TNT‐specific markers and combine tissue clearing, super‐resolution imaging and molecular tracing to determine more conclusively whether these structures represent bona fide TNTs in vivo and to visualize TNT‐mediated lysosome transport in bone. Together, our findings support the presence of TNTs in the osteocyte network in vivo.

We next asked whether osteocytic TNTs share canonical TNT features while exhibiting osteocyte‐specific characteristics. Consistent with TNTs reported across cell types [17, 57, 58, 59, 60], TNTs in MLO‐Y4 osteocytes were highly dynamic, nanometre‐thin, variably long, largely unbranched and open‐ended connections, with α‐tubulin present in a subset (Figure 1). TNTs formed by protrusion elongation or cell dislodgement (Figure 1), as reported in PC12 cells [61]. These properties support their identification as bona fide TNTs. Furthermore, osteocytic TNTs also exhibited distinct cell‐type specificity arising from the unique dendritic architecture. TNTs formed predominantly between dendrite‐dendrite and dendrite‐cell body pairs, whereas cell body‐cell body connections occurred only infrequently (Figure 2A–G). This distribution pattern may reflect the elaborate dendritic network of osteocytes, which could increase TNT formation via cell dislodgement following dendritic contact with neighbouring cells. Furthermore, TNTs connecting cell bodies were thicker and longer than TNTs involving dendritic connections (Figure 2G), suggesting that the connection site may influence TNT structure and potentially function.

Interestingly, recent work by Chang et al. [62] identified dendrite‐dendrite intercellular structures between cortical neurons, termed dendrite‐dendrite nanotubes (DNTs), which are likewise highly dynamic and primarily F‐actin–based. Remarkably, the mean diameter of DNTs (~0.34 μm) is nearly identical to that of dendrite‐dendrite TNTs between osteocytes (0.35 μm) (Figure 2G). This finding suggests that the two structures may share certain morphological similarities. Such a comparison also has a biological basis, as osteocytes and neurons share commonalities in gene expression profiles, complex dendritic morphology and highly specialized intercellular communication networks [52, 63]. However, due to the current lack of molecular markers that can definitively distinguish DNTs from TNTs, it remains premature to classify osteocytic dendrite‐dendrite nanotubes as DNTs. Thus, whether osteocytic dendrite‐dendrite nanotubes represent a specialized TNT subtype, an osteocytic analogue of DNTs or a hybrid structure remains to be clarified.

Cx43 is highly enriched in osteocyte dendrites and is typically associated with the transmission of electrical signals or small molecules (< 1 kDa) through gap junction channels [64]. Here, we found that a subset of osteocytic TNTs also expressed Cx43, predominantly at their termini (Figure 2H–J). This specific spatial pattern suggests that Cx43 may be involved in anchoring TNTs to the target cell membrane and in stabilizing these connections [43, 65, 66]. Cx43 enrichment at TNT ends has been linked to electrical coupling and proposed as a compensatory route when gap junctional signalling is compromised [67, 68]. Although whether osteocytic TNTs serve a similar role remains unclear, terminal Cx43 could provide an auxiliary coupling mechanism alongside dendritic Cx43‐based gap junctions in osteocytes. In other contexts, BMSCs can transfer Cx43 to cardiomyocytes via TNTs, thereby alleviating isoproterenol‐induced cardiac hypertrophy [69]. However, the localization pattern of Cx43 in osteocytic TNTs favours a role as a structural or functional component of the TNT itself rather than a cargo being transported. Notably, Cx43 was present in only a subset of osteocytic TNTs and did not correlate with TNT diameter or length (Figure 2H,I), suggesting that Cx43 is dispensable for TNT formation and that Cx43^−^ TNTs may represent a distinct communication route. Whether Cx43^+^ and Cx43^−^ TNTs support different signalling modalities and how Cx43 functions within Cx43^+^ TNTs, remain to be determined.

Stressful microenvironments have been shown to induce TNT formation [43]. Here, oxidative stress, serum starvation and acidic environments all significantly promote the formation of osteocytic TNTs (Figure 2K–P). Although the pathways that link specific stress signals to TNT biogenesis remain undefined, TNT induction may represent an adaptive response that supports cell survival under adverse conditions [58]. Within the bone microenvironment, osteocytes rely primarily on interstitial fluid for nutrient delivery. Consequently, perfusion or nutrient deficits—which may result from microdamage, oestrogen deficiency or other pathological conditions—are major drivers of osteocyte dysfunction and apoptosis [70]. Accordingly, we used serum starvation as an in vitro nutrient‐deprivation model for subsequent studies. It should be noted that serum starvation still represents a relatively simplified in vitro model and is therefore insufficient to fully recapitulate the nutrient microenvironment experienced by osteocytes in bone tissue. Nevertheless, this model remains useful for assessing the cytoprotective role of TNTs under nutrient deprivation‐related stress conditions, although the conclusions drawn from it still require further validation in models that more closely reflect the in vivo microenvironment.

The present study corroborated that osteocytic TNTs can effectively mediate cargo transfer, fulfilling a functional criterion of bona fide TNTs. In co‐culture, almost no transfer of EGFP (representing cytoplasmic proteins) or CMTPX (labelling the cytoplasmic matrix) was detected (Figure 3A,B). Instead, abundant DiD^+^ cargo trafficked along TNTs, suggesting that osteocytes selectively transfer membrane‐bound cargo rather than bulk cytoplasmic components through TNTs. This cargo selectivity aligns with observations in diverse cell types, although the mechanisms governing TNT cargo specificity remain unclear [58]. In osteocytes, membrane‐bound cargo appears to constitute the principal functional cargo transferred via TNTs. Consistent with this idea, membrane‐bound cargo transfer via TNTs has been implicated in diverse contexts, including neuronal signalling and stromal support of leukaemia cells [21, 71]. Our findings extend these observations to osteocytes by showing that nutrient deprivation promotes TNT formation (Figure 2O,P) and enhances cargo transfer (Figure 4A–C), which effectively alleviates stress‐induced apoptosis in recipient osteocytes (Figure 4D–G).

To further define the membrane‐bound cargo transferred via TNTs, we analysed the intracellular sorting behaviour of DiD. Lipophilic carbocyanine dyes are routed intracellularly largely according to tail‐chain chemistry [46, 72]. Long, saturated tails tend to accumulate in late endosomes/lysosomes, whereas shorter or unsaturated tails preferentially enter recycling pathways [46, 72]. DiD (DiIC_18_(5)), utilized in this study, contains two saturated octadecyl chains and is therefore expected to accumulate in the lysosomal compartment following internalization. Consistent with this prediction, LysoTracker signals showed clear colocalization with DiD^+^ cargo within TNTs. The mean values of Pearson's r and Manders' M1 both fell within the 0.7–0.8 range, whereas Manders' M2 was close to 1, indicating that most DiD^+^ cargo corresponded to lysosome‐related compartments and that nearly all LysoTracker‐positive signals were encompassed by DiD signals (Figure 5C–F). Flow cytometric quantification and CLSM imaging further corroborated the substantial intercellular transfer of lysosomes via TNTs (Figure 5G–J). Although DiD is not lysosome‐specific, the combined evidence—including DiD's lipid‐sorting behaviour, its extensive colocalization with LysoTracker and the cytoprotective effect of TNT‐mediated lysosome transfer—supports the conclusion that lysosomes are the predominant DiD^+^ membrane‐bound cargo transferred between osteocytes. In addition to lysosomes, we also detected mitochondrial transfer through TNTs (Figure 5). However, only lysosome transfer exerted a cytoprotective effect, while mitochondrial transfer did not mitigate apoptosis (Figure S5). Although mitochondrial transfer via TNTs has been reported in other cell types and implicated in processes such as mitochondrial quality control and metabolic homeostasis, its biological significance in osteocytes remains to be determined [73].

We then investigated the functional significance of intercellular lysosome transfer between osteocytes. Autophagy is a pivotal process for maintaining cellular homeostasis, and its function depends on intact autophagic flux [74]. Impaired autophagosome–lysosome fusion blocks autophagic flux and jeopardizes cell survival [75]. In the present study, we found that starvation stress induced autophagic flux impairment in osteocytes. In contrast, healthy osteocytes delivered functional lysosomes to stressed osteocytes via TNTs, restoring autophagic flux in the recipient cells (Figure 6A–N). These findings define a previously unrecognized mode of ‘transcellular autophagy’, driven by TNT‐mediated lysosome sharing. Notably, ‘transcellular autophagy’ was recently proposed by Wang et al. [76] in a hepatocyte model, in which damaged hepatocytes transfer autophagosomes to adjacent healthy hepatocytes via TNTs for degradation, thus alleviating autophagic flux blockade in damaged cells. Our study unveils an alternative form of transcellular autophagy, mediated not by autophagosome transfer but by ‘lysosome sharing’. In this mechanism, healthy osteocytes directly provide the essential degradation organelles—lysosomes—via TNTs to neighbouring stressed osteocytes with impaired autophagy, thereby restoring autophagic flux and suppressing apoptosis. This finding suggests that different cell types may utilize distinct transcellular autophagy strategies to rescue compromised cells and preserve tissue homeostasis [77, 78]. In osteocytes, this rescue mechanism is achieved specifically through TNT‐mediated lysosome sharing (Figure 6P,Q).

How restoration of autophagic flux suppresses osteocyte apoptosis remains unclear. However, its potential downstream mechanisms may be at least partly associated with the attenuation of intracellular pro‐apoptotic signalling. First, restoration of autophagic flux may enhance the clearance of damaged mitochondria, thereby reducing reactive oxygen species (ROS) accumulation, alleviating BAX/BAK‐mediated mitochondrial outer membrane permeabilization, decreasing cytochrome c release and consequently suppressing subsequent caspase‐9 and caspase‐3 activation [79, 80]. Second, as p62 accumulation is alleviated, the activation of caspase‐8‐associated pro‐apoptotic signalling may also be restrained [81]. Beyond these processes, the anti‐apoptotic effect of restored autophagic flux may also involve additional signalling pathways. These downstream events were not directly examined in the present study, and further studies are required to elucidate the precise molecular mechanisms by which TNT‐mediated lysosome sharing suppresses apoptosis in osteocytes.

## 5 Conclusion

In summary, this study provides the first systematic identification of TNT structures between cultured osteocytes and demonstrates that they mediate a previously unrecognized form of transcellular autophagy through intercellular lysosomal sharing. This mechanism helps preserve osteocyte viability under stress. Our findings expand current understanding of how osteocytes maintain population survival under both physiological and pathological conditions. Although our in vitro evidence strongly supports this lysosome‐sharing–based rescue mechanism, several key questions remain. Future studies should establish more stringent in vivo labelling and tracking approaches, combined with functional perturbations, to determine whether this mechanism operates within the LCS and to define its contribution to osteocyte survival and bone homeostasis. In parallel, it will be essential to investigate whether osteocytic TNTs transfer additional cargoes (e.g., Ca^2+^, proteins or other organelles) and how TNT‐mediated transfer intersects with Cx43‐mediated gap junction communication. Further delineation of the molecular mechanisms governing TNT formation, cargo selection and functional outcomes will provide a foundation for mechanism‐based strategies to modulate this communication axis. Osteocytic TNTs and the lysosome‐sharing–mediated transcellular autophagy pathway they facilitate may represent a new therapeutic entry point. Targeting this axis could offer novel strategies to restore bone homeostasis and treat bone diseases such as osteoporosis.

## Author Contributions

Conceptualization: Jinbiao Qiang and Ce Shi. Formal analysis: Jinbiao Qiang. Funding acquisition: Ce Shi. Investigation: Jinbiao Qiang, Ronghao Jin, Fang Zheng and Jinwei Li. Methodology: Jinbiao Qiang, Ronghao Jin, Tong Sha, Yijun Zhou, Yue Hu, Shuyu Zhang, Zhenming Yang, Mengdong Nie, Huanyu Luo, Xiaoduo Tang, Hao Guo, Cangwei Liu and Ce Shi. Project administration: Jinbiao Qiang and Ce Shi. Resources: Tong Sha. Supervision: Yue Hu, Hongchen Sun, Cangwei Liu and Ce Shi. Validation: Jinbiao Qiang. Visualization: Jinbiao Qiang, Tong Sha and Zunxuan Xie. Writing – original draft preparation: Jinbiao Qiang. Writing – review and editing: Jinbiao Qiang, Fang Zheng, Cangwei Liu and Ce Shi.

## Funding

This work was supported by the National Natural Science Foundation of China (82270959). The Science and Technology Project of Jilin Provincial Department of Finance (JCSZ2025678‐1, JCSZ2023481‐34, JCSZ2021893‐36). Fundamental Research Funds for the Central Universities.

## Conflicts of Interest

The authors declare no conflicts of interest.

## Supporting information


**Figure S1:** Identification of primary osteocytes from mice and humans. (A) Representative ALP staining images of mouse primary osteoblasts (digest 5) and osteocytes (digest 9). Scale bar, 20 μm. (B) Relative mRNA expression levels of osteocyte‐specific markers *E11* and *Dmp1* in mouse primary osteoblasts (digest 5), and osteocytes (digest 9) (*n* = 3). (C) Representative ALP staining images of human primary osteoblasts (digest 5) and osteocytes (digest 9). Scale bar, 20 μm. (D) Relative mRNA expression levels of osteocyte‐specific markers *E11* and *DMP1* in human primary osteoblasts (digest 5) and osteocytes (digest 9) (*n* = 3). Data are presented as mean ± SE. Statistical significance was assessed using an unpaired two‐tailed Student's *t*‐test. **p < 0.01, ***p < 0.001.
**Figure S2:** Effects of cytochalasin B and oxidative stress on cell viability. (A) Effects of cytochalasin B at different concentrations (0, 0.25, 0.5 and 1 μM) on cell viability (*n* = 3). (B) Effects of 100 and 200 μM H_2_O_2_ treatment for 2 h on cell viability (*n* = 3). (C) Effects of treatment with 100 or 200 μM H_2_O_2_ for 24 h on cell viability (*n* = 3). Data are presented as mean ± SE. Statistical significance was assessed using one‐way ANOVA with Tukey's post hoc test.
**Figure S3:** TNT‐mediated transport of membrane‐bound cargo between MLO‐Y4 cells. Live‐cell imaging of the transport of DiD‐labelled membrane‐bound cargo between MLO‐Y4 cells via TNTs. Arrows indicate DiD‐labelled cargo moving along a TNT. Scale bar, 5 μm.
**Figure S4:** Lysosome transport via TNTs may rescue dying osteocytes. Representative CLSM image of lysosome transport via TNTs between normal and dying osteocytes. White arrows indicate dying osteocytes and yellow arrows indicate lysosomes transported within TNTs. Scale bar, 20 μm.
**Figure S5:** Mitochondrial transfer via TNTs does not restore autophagic flux or prevent apoptosis in osteocytes. (A) Schematic diagram of the co‐culture and FACS strategy using normally cultured MitoTracker‐labelled donor cells (green) co‐cultured with CMTPX‐labelled recipient cells (red) under serum starvation, with or without BafA1 treatment. FACS gates: CMTPX⁻/MitoTracker⁺ cells (donor cells), CMTPX⁺/MitoTracker⁺ cells (MitoTracker⁺ recipient cells) and CMTPX⁺/MitoTracker⁻ cells (MitoTracker⁻ recipient cells). (B) Western blot analysis of autophagy‐related proteins in donor cells after FACS. (C, D) Semi‐quantification of LC3‐II (C) and p62 (D) protein levels in donor cells (*n* = 3). (E) Western blot analysis of autophagy‐related proteins in recipient cells after FACS. (F, G) Semi‐quantification of LC3‐II (F) and p62 (G) protein levels in recipient cells (*n* = 3). (H, I) Flow cytometry plots of Annexin V signals in recipient cells (H) and quantification of the percentage of Annexin V‐positive cells in recipient cells (I) (*n* = 3). Data are presented as mean ± SE. Statistical significance was assessed using one‐way ANOVA with Tukey's post hoc test for multiple group comparisons and an unpaired two‐tailed Student's *t*‐test for two‐group comparisons. *p < 0.05, **p < 0.01; ns, not significant.
**Table S1:** Primer sequences for qRT‐PCR.

**Movie S1:** Time‐lapse recording of DiD‐labelled membrane‐bound cargo transported between MLO‐Y4 osteocytes via TNTs.

## Data Availability

The data that support the findings of this study are available from the corresponding author upon reasonable request.

## References

[1] Q. Zhu , Y. Fu , C. P. Cui , et al., “OTUB1 Promotes Osteoblastic Bone Formation Through Stabilizing FGFR2,” Signal Transduction and Targeted Therapy 8, no. 1 (2023): 142, 10.1038/s41392-023-01354-2. 37024477 PMC10079838

[2] S. Stegen and G. Carmeliet , “Metabolic Regulation of Skeletal Cell Fate and Function,” Nature Reviews. Endocrinology 20, no. 7 (2024): 399–413, 10.1038/s41574-024-00969-x. 38499689

[3] J. Delgado‐Calle and T. Bellido , “The Osteocyte as a Signaling Cell,” Physiological Reviews 102, no. 1 (2022): 379–410, 10.1152/physrev.00043.2020. 34337974 PMC8858675

[4] Z. Jiang , G. Qi , X. He , et al., “Ferroptosis in Osteocytes as a Target for Protection Against Postmenopausal Osteoporosis,” Advanced Science 11, no. 12 (2024): e2307388, 10.1002/advs.202307388. 38233202 PMC10966575

[5] S. L. Dallas , M. Prideaux , and L. F. Bonewald , “The Osteocyte: An Endocrine Cell … and More,” Endocrine Reviews 34, no. 5 (2013): 658–690, 10.1210/er.2012-1026. 23612223 PMC3785641

[6] S. Ding , Y. Chen , C. Huang , L. Song , Z. Liang , and B. Wei , “Perception and Response of Skeleton to Mechanical Stress,” Physics of Life Reviews 49 (2024): 77–94, 10.1016/j.plrev.2024.03.011. 38564907

[7] R. L. Jilka and C. A. O'Brien , “The Role of Osteocytes in Age‐Related Bone Loss,” Current Osteoporosis Reports 14, no. 1 (2016): 16–25, 10.1007/s11914-016-0297-0. 26909563

[8] J. Y. Ru and Y. F. Wang , “Osteocyte Apoptosis: The Roles and Key Molecular Mechanisms in Resorption‐Related Bone Diseases,” Cell Death & Disease 11, no. 10 (2020): 846, 10.1038/s41419-020-03059-8. 33046704 PMC7552426

[9] J. Gao , A. Qin , D. Liu , et al., “Endoplasmic Reticulum Mediates Mitochondrial Transfer Within the Osteocyte Dendritic Network,” Science Advances 5, no. 11 (2019): eaaw7215, 10.1126/sciadv.aaw7215. 31799389 PMC6867880

[10] J. Su , Y. Song , Z. Zhu , et al., “Cell‐Cell Communication: New Insights and Clinical Implications,” Signal Transduction and Targeted Therapy 9, no. 1 (2024): 196, 10.1038/s41392-024-01888-z. 39107318 PMC11382761

[11] B. A. Yang , T. M. Westerhof , K. Sabin , S. D. Merajver , and C. A. Aguilar , “Engineered Tools to Study Intercellular Communication,” Advanced Science (Weinheim, Germany) 8, no. 3 (2021): 2002825, 10.1002/advs.202002825. PMC7856891 33552865

[12] L. Miao , Y. Lu , A. Nusrat , et al., “Tunneling Nanotube‐Like Structures Regulate Distant Cellular Interactions During Heart Formation,” Science 387, no. 6739 (2025): eadd3417, 10.1126/science.add3417. 40080583 PMC12662173

[13] R. Hua , V. A. Truong , R. J. Fajardo , T. Guda , S. Gu , and J. X. Jiang , “Connexin Hemichannels Drive Lactation‐Induced Osteocyte Acidification and Perilacunar‐Canalicular Remodeling,” Cell Reports 43, no. 7 (2024): 114363, 10.1016/j.celrep.2024.114363. 38935505 PMC11318086

[14] X. Liu , M. Bai , Y. Sun , et al., “FGF7‐Induced E11 Facilitates Cell‐Cell Communication Through connexin43,” International Journal of Biological Sciences 17, no. 14 (2021): 3862–3874, 10.7150/ijbs.65240. 34671204 PMC8495393

[15] M. A. Riquelme , S. Gu , R. Hua , and J. X. Jiang , “Mechanotransduction via the Coordinated Actions of Integrins, PI3K Signaling and Connexin Hemichannels,” Bone Research 9, no. 1 (2021): 8, 10.1038/s41413-020-00126-w. 33531460 PMC7854719

[16] E. Y. Kirichenko , S. N. Skatchkov , and A. M. Ermakov , “Structure and Functions of Gap Junctions and Their Constituent Connexins in the Mammalian CNS,” Biochemistry (Moscow), Supplement Series A: Membrane and Cell Biology 15, no. 2 (2021): 107–119, 10.1134/s1990747821020069. 34512926 PMC8432592

[17] A. Rustom , R. Saffrich , I. Markovic , P. Walther , and H. H. Gerdes , “Nanotubular Highways for Intercellular Organelle Transport,” Science 303, no. 5660 (2004): 1007–1010, 10.1126/science.1093133. 14963329

[18] J. G. Baldwin , C. Heuser‐Loy , T. Saha , et al., “Intercellular Nanotube‐Mediated Mitochondrial Transfer Enhances T Cell Metabolic Fitness and Antitumor Efficacy,” Cell 187, no. 23 (2024): 6614–6630.e6621, 10.1016/j.cell.2024.08.029. 39276774 PMC11623344

[19] H. Scheiblich , C. Dansokho , D. Mercan , et al., “Microglia Jointly Degrade Fibrillar Alpha‐Synuclein Cargo by Distribution Through Tunneling Nanotubes,” Cell 184, no. 20 (2021): 5089–5106.e5021, 10.1016/j.cell.2021.09.007. 34555357 PMC8527836

[20] R. Z. Lin , G. B. Im , A. C. Luo , et al., “Mitochondrial Transfer Mediates Endothelial Cell Engraftment Through Mitophagy,” Nature 629, no. 8012 (2024): 660–668, 10.1038/s41586-024-07340-0. 38693258 PMC11574736

[21] M. D. Kolba , W. Dudka , M. Zaręba‐Kozioł , et al., “Tunneling Nanotube‐Mediated Intercellular Vesicle and Protein Transfer in the Stroma‐Provided Imatinib Resistance in Chronic Myeloid Leukemia Cells,” Cell Death & Disease 10, no. 11 (2019): 817, 10.1038/s41419-019-2045-8. 31659149 PMC6817823

[22] H. Scheiblich , F. Eikens , L. Wischhof , et al., “Microglia Rescue Neurons From Aggregate‐Induced Neuronal Dysfunction and Death Through Tunneling Nanotubes,” Neuron 112, no. 18 (2024): 3106–3125.e3108, 10.1016/j.neuron.2024.06.029. 39059388

[23] X. Zheng , J. Wang , H. Zhou , et al., “Dental Pulp Stem Cells Alleviate Schwann Cell Pyroptosis via Mitochondrial Transfer to Enhance Facial Nerve Regeneration,” Bioactive Materials 47 (2025): 313–326, 10.1016/j.bioactmat.2025.01.031. 40026822 PMC11869962

[24] V. Venkataramani , M. Schneider , F. A. Giordano , et al., “Disconnecting Multicellular Networks in Brain Tumours,” Nature Reviews. Cancer 22, no. 8 (2022): 481–491, 10.1038/s41568-022-00475-0. 35488036

[25] S. C. Watkins and R. D. Salter , “Functional Connectivity Between Immune Cells Mediated by Tunneling Nanotubules,” Immunity 23, no. 3 (2005): 309–318, 10.1016/j.immuni.2005.08.009. 16169503

[26] J. Che , Y. Cheng , X. Wu , Z. Wu , L. Shang , and Y. Zhao , “M‐Sec Overexpressed Stem Cells Growing Abundant Tunneling Nanotubes for Diverse Active Transferring,” ACS Nano 19, no. 31 (2025): 28644–28659, 10.1021/acsnano.5c08001. 40743507

[27] J. J. Neher and M. Simons , “Protective Lifelines: Tunneling Nanotubes Connect Neurons and Microglia,” Neuron 112, no. 18 (2024): 2991–2993, 10.1016/j.neuron.2024.07.027. 39326386

[28] R. Chakraborty , T. Nonaka , M. Hasegawa , and C. Zurzolo , “Tunnelling Nanotubes Between Neuronal and Microglial Cells Allow Bi‐Directional Transfer of α‐Synuclein and Mitochondria,” Cell Death & Disease 14, no. 5 (2023): 329, 10.1038/s41419-023-05835-8. 37202391 PMC10195781

[29] A. Sartori‐Rupp , D. Cordero Cervantes , A. Pepe , et al., “Correlative Cryo‐Electron Microscopy Reveals the Structure of TNTs in Neuronal Cells,” Nature Communications 10, no. 1 (2019): 342, 10.1038/s41467-018-08178-7. PMC6341166 30664666

[30] J. Q. Zhang , A. Takahashi , J. Y. Gu , et al., “In Vitro and In Vivo Detection of Tunneling Nanotubes in Normal and Pathological Osteoclastogenesis Involving Osteoclast Fusion,” Laboratory Investigation 101, no. 12 (2021): 1571–1584, 10.1038/s41374-021-00656-9. 34537825

[31] S. Sagar , M. I. Faizan , N. Chaudhary , et al., “Obesity Impairs Cardiolipin‐Dependent Mitophagy and Therapeutic Intercellular Mitochondrial Transfer Ability of Mesenchymal Stem Cells,” Cell Death & Disease 14, no. 5 (2023): 324, 10.1038/s41419-023-05810-3. 37173333 PMC10181927

[32] J. Jin and P. A. Nolte , “Mitochondrial Distribution and Osteocyte Mechanosensitivity,” Current Osteoporosis Reports 23, no. 1 (2025): 22, 10.1007/s11914-025-00918-1. 40402395 PMC12098195

[33] H. Gunasekara , Y. S. Cheng , V. Perez‐Silos , et al., “Unveiling Cellular Communications Through Rapid Pan‐Membrane‐Protein Labeling,” Nature Communications 16, no. 1 (2025): 3584, 10.1038/s41467-025-58779-2. PMC12000395 40234465

[34] A. R. Stern , M. M. Stern , M. E. Van Dyke , K. Jähn , M. Prideaux , and L. F. Bonewald , “Isolation and Culture of Primary Osteocytes From the Long Bones of Skeletally Mature and Aged Mice,” BioTechniques 52, no. 6 (2012): 361–373, 10.2144/0000113876. 22668415 PMC3612989

[35] S. Motoike , Y. Inada , J. Toguchida , M. Kajiya , and M. Ikeya , “Jawbone‐Like Organoids Generated From Human Pluripotent Stem Cells,” Nature Biomedical Engineering 9 (2025): 1816–1834, 10.1038/s41551-025-01419-3. PMC12623242 40603745

[36] I. Sáenz‐de‐Santa‐María , J. M. Henderson , A. Pepe , and C. Zurzolo , “Identification and Characterization of Tunneling Nanotubes for Intercellular Trafficking,” Current Protocols 3, no. 11 (2023): e939, 10.1002/cpz1.939. 37994667

[37] K. McCoy‐Simandle , S. J. Hanna , and D. Cox , “Exosomes and Nanotubes: Control of Immune Cell Communication,” International Journal of Biochemistry & Cell Biology 71 (2016): 44–54, 10.1016/j.biocel.2015.12.006. 26704468 PMC4720554

[38] T. Shi , S. Shen , Y. Shi , et al., “Osteocyte‐Derived Sclerostin Impairs Cognitive Function During Ageing and Alzheimer's Disease Progression,” Nature Metabolism 6, no. 3 (2024): 531–549, 10.1038/s42255-024-00989-x. 38409606

[39] J. Kaur , M. Adhikari , H. M. Sabol , et al., “Single‐Cell Transcriptomic Analysis Identifies Senescent Osteocytes That Trigger Bone Destruction in Breast Cancer Metastasis,” Cancer Research 84, no. 23 (2024): 3936–3952, 10.1158/0008-5472.Can-24-0857. 39312185 PMC11611663

[40] N. Liu , Y. Ma , W. Gong , et al., “Osteocyte‐Derived Extracellular Vesicles Mediate the Bone‐To‐Cartilage Crosstalk and Promote Osteoarthritis Progression,” Nature Communications 16, no. 1 (2025): 4746, 10.1038/s41467-025-59861-5. PMC12095588 40399261

[41] R. Wang , B. Mehrjou , D. Dehghan‐Banian , et al., “Targeting Long Noncoding RNA H19 in Subchondral Bone Osteocytes and the Alleviation of Cartilage Degradation in Osteoarthritis,” Arthritis & Rhematology 77, no. 3 (2025): 283–297, 10.1002/art.43028. PMC11865692 39482250

[42] T. Pan , F. Liu , X. Hao , et al., “BIGH3 Mediates Apoptosis and Gap Junction Failure in Osteocytes During Renal Cell Carcinoma Bone Metastasis Progression,” Cancer Letters 596 (2024): 217009, 10.1016/j.canlet.2024.217009. 38849015 PMC11964150

[43] F. Palese , M. Rakotobe , and C. Zurzolo , “Transforming the Concept of Connectivity: Unveiling Tunneling Nanotube Biology and Their Roles in Brain Development and Neurodegeneration,” Physiological Reviews 105, no. 3 (2025): 1823–1865, 10.1152/physrev.00023.2024. 40067081

[44] F. Guan , X. Wu , J. Zhou , et al., “Mitochondrial Transfer in Tunneling Nanotubes‐A New Target for Cancer Therapy,” Journal of Experimental & Clinical Cancer Research 43, no. 1 (2024): 147, 10.1186/s13046-024-03069-w. 38769583 PMC11106947

[45] D. Cordero Cervantes and C. Zurzolo , “Peering Into Tunneling Nanotubes‐The Path Forward,” EMBO Journal 40, no. 8 (2021): e105789, 10.15252/embj.2020105789. 33646572 PMC8047439

[46] S. Mukherjee , T. T. Soe , and F. R. Maxfield , “Endocytic Sorting of Lipid Analogues Differing Solely in the Chemistry of Their Hydrophobic Tails,” Journal of Cell Biology 144, no. 6 (1999): 1271–1284, 10.1083/jcb.144.6.1271. 10087269 PMC2150570

[47] Z. Yu , S. Lin , X. Gong , et al., “The Role of Macroautophagy in Substance Use Disorders,” Annals of the New York Academy of Sciences 1543, no. 1 (2025): 68–78, 10.1111/nyas.15272. 39714908

[48] D. Liu , Y. Gao , J. Liu , et al., “Intercellular Mitochondrial Transfer as a Means of Tissue Revitalization,” Signal Transduction and Targeted Therapy 6, no. 1 (2021): 65, 10.1038/s41392-020-00440-z. 33589598 PMC7884415

[49] F. Barutta , S. Bellini , S. Kimura , et al., “Protective Effect of the Tunneling Nanotube‐TNFAIP2/M‐Sec System on Podocyte Autophagy in Diabetic Nephropathy,” Autophagy 19, no. 2 (2023): 505–524, 10.1080/15548627.2022.2080382. 35659195 PMC9851239

[50] G. Pinto , C. Brou , and C. Zurzolo , “Tunneling Nanotubes: The Fuel of Tumor Progression?,” Trends Cancer 6, no. 10 (2020): 874–888, 10.1016/j.trecan.2020.04.012. 32471688

[51] L. Heinke , “Connecting Cells Through TNT,” Nature Reviews. Molecular Cell Biology 26, no. 1 (2025): 6, 10.1038/s41580-024-00811-2. 39558025

[52] R. M. Guerra , V. M. Fowler , and L. Wang , “Osteocyte Dendrites: How Do They Grow, Mature, and Degenerate in Mineralized Bone?,” Cytoskeleton (Hoboken) 82, no. 9 (2025): 556–570, 10.1002/cm.21964. 39651620 PMC12146430

[53] P. R. Buenzli and N. A. Sims , “Quantifying the Osteocyte Network in the Human Skeleton,” Bone 75 (2015): 144–150, 10.1016/j.bone.2015.02.016. 25708054

[54] N. K. Wittig , M. Laugesen , M. E. Birkbak , et al., “Canalicular Junctions in the Osteocyte Lacuno‐Canalicular Network of Cortical Bone,” ACS Nano 13, no. 6 (2019): 6421–6430, 10.1021/acsnano.8b08478. 31095362

[55] O. Korenkova , S. Liu , I. Prlesi , et al., “Tunneling Nanotubes Enable Intercellular Transfer in Zebrafish Embryos,” Developmental Cell 60, no. 4 (2025): 524–534.e523, 10.1016/j.devcel.2024.10.016. 39541978

[56] M. Tilton , J. Liao , C. Kim , et al., “Tracing Cellular Senescence in Bone: Time‐Dependent Changes in Osteocyte Cytoskeleton Mechanics and Morphology,” Small 21, no. 14 (2025): e2408517, 10.1002/smll.202408517. 40026102 PMC11985287

[57] J. Yuan , F. Chen , D. Jiang , Z. Xu , H. Zhang , and Z. B. Jin , “ROCK Inhibitor Enhances Mitochondrial Transfer via Tunneling Nanotubes in Retinal Pigment Epithelium,” Theranostics 14, no. 15 (2024): 5762–5777, 10.7150/thno.96508. 39346535 PMC11426248

[58] C. Zhu , Y. Shi , and J. You , “Immune Cell Connection by Tunneling Nanotubes: The Impact of Intercellular Cross‐Talk on the Immune Response and Its Therapeutic Applications,” Molecular Pharmaceutics 18, no. 3 (2021): 772–786, 10.1021/acs.molpharmaceut.0c01248. 33529022

[59] M. Rakotobe and C. Zurzolo , “Beyond Synapses: Cytoplasmic Connections in Brain Function and Evolution,” Biological Reviews of the Cambridge Philosophical Society 100, no. 5 (2025): 2055–2070, 10.1111/brv.70034. 40515735 PMC12407032

[60] P. K. Melwani and B. N. Pandey , “Tunneling Nanotubes: The Intercellular Conduits Contributing to Cancer Pathogenesis and Its Therapy,” Biochimica Et Biophysica Acta. Reviews on Cancer 1878, no. 6 (2023): 189028, 10.1016/j.bbcan.2023.189028. 37993000

[61] N. V. Bukoreshtliev , X. Wang , E. Hodneland , S. Gurke , J. F. Barroso , and H. H. Gerdes , “Selective Block of Tunneling Nanotube (TNT) Formation Inhibits Intercellular Organelle Transfer Between PC12 Cells,” FEBS Letters 583, no. 9 (2009): 1481–1488, 10.1016/j.febslet.2009.03.065. 19345217

[62] M. Chang , S. Krüssel , L. K. Parajuli , et al., “Intercellular Communication in the Brain Through a Dendritic Nanotubular Network,” Science 390, no. 6768 (2025): eadr7403, 10.1126/science.adr7403. 41037599

[63] J. S. Wang , T. Kamath , C. M. Mazur , et al., “Control of Osteocyte Dendrite Formation by Sp7 and Its Target Gene Osteocrin,” Nature Communications 12, no. 1 (2021): 6271, 10.1038/s41467-021-26571-7. PMC8560803 34725346

[64] R. Hua , J. Zhang , M. A. Riquelme , and J. X. Jiang , “Connexin Gap Junctions and Hemichannels Link Oxidative Stress to Skeletal Physiology and Pathology,” Current Osteoporosis Reports 19, no. 1 (2021): 66–74, 10.1007/s11914-020-00645-9. 33403446 PMC8174533

[65] S. A. Lucaciu , S. E. Leighton , A. Hauser , R. Yee , and D. W. Laird , “Diversity in Connexin Biology,” Journal of Biological Chemistry 299, no. 11 (2023): 105263, 10.1016/j.jbc.2023.105263. 37734551 PMC10598745

[66] S. Sinha , B. W. Callow , A. P. Farfel , et al., “Breast Cancers That Disseminate to Bone Marrow Acquire Aggressive Phenotypes Through CX43‐Related Tumor‐Stroma Tunnels,” Journal of Clinical Investigation 134, no. 24 (2024): e170953, 10.1172/jci170953. 39480488 PMC11645149

[67] X. Wang , M. L. Veruki , N. V. Bukoreshtliev , E. Hartveit , and H. H. Gerdes , “Animal Cells Connected by Nanotubes Can Be Electrically Coupled Through Interposed Gap‐Junction Channels,” Proceedings of the National Academy of Sciences of the United States of America 107, no. 40 (2010): 17194–17199, 10.1073/pnas.1006785107. 20855598 PMC2951457

[68] V. Mulchandani , A. Banerjee , A. V. Vadlamannati , S. Kumar , and J. Das Sarma , “Connexin 43 Trafficking and Regulation of Gap Junctional Intercellular Communication Alters Ovarian Cancer Cell Migration and Tumorigenesis,” Biomedicine & Pharmacotherapy 159 (2023): 114296, 10.1016/j.biopha.2023.114296. 36701988

[69] J. Zhang , H. Jiang , S. Liu , et al., “Bone Marrow Mesenchymal Stem Cells Transport connexin43 via Tunneling Nanotubes to Alleviate Isopreterenol‐Induced Myocardial Hypertrophy,” Stem Cell Res Ther 16, no. 1 (2025): 229, 10.1186/s13287-025-04339-w. 40329337 PMC12057053

[70] S. A. Al‐Dujaili , E. Lau , H. Al‐Dujaili , K. Tsang , A. Guenther , and L. You , “Apoptotic Osteocytes Regulate Osteoclast Precursor Recruitment and Differentiation In Vitro,” Journal of Cellular Biochemistry 112, no. 9 (2011): 2412–2423, 10.1002/jcb.23164. 21538477

[71] S. Bhat , N. Ljubojevic , S. Zhu , M. Fukuda , A. Echard , and C. Zurzolo , “Rab35 and Its Effectors Promote Formation of Tunneling Nanotubes in Neuronal Cells,” Scientific Reports 10, no. 1 (2020): 16803, 10.1038/s41598-020-74013-z. 33033331 PMC7544914

[72] L. Rajendran , H.‐J. Knölker , and K. Simons , “Subcellular Targeting Strategies for Drug Design and Delivery,” Nature Reviews Drug Discovery 9, no. 1 (2010): 29–42, 10.1038/nrd2897. 20043027

[73] Y. Liu , W. L. Dissanayaka , and C. Yiu , “Therapeutic Implications of Mitochondrial Transfer on Stem Cell Fate in Regenerative Medicine,” Journal of Translational Medicine 23, no. 1 (2025): 568, 10.1186/s12967-025-06472-9. 40399970 PMC12093763

[74] J. Gan , R. Zhao , D. Zheng , et al., “PYGM Protects Against Myocardial Infarction by Enhancing Glycogenolysis and Facilitating Autophagic Flux,” Circulation 152 (2025): 1146–1165, 10.1161/circulationaha.124.072312. 40988610

[75] Q. Zhang , S. Cao , F. Qiu , and N. Kang , “Incomplete Autophagy: Trouble Is a Friend,” Medicinal Research Reviews 42, no. 4 (2022): 1545–1587, 10.1002/med.21884. 35275411

[76] T. Wang , L. Wang , J. Sun , et al., “Tunneling Nanotube‐Mediated Transcellular Autophagy Alleviates Cadmium Induced Hepatocyte Injury,” Advanced Science (Weinheim) 12, no. 37 (2025): e02793, 10.1002/advs.202502793. PMC12499425 40719264

[77] V. Karthik and A. R. Guntur , “Energy Metabolism of Osteocytes,” Current Osteoporosis Reports 19, no. 4 (2021): 444–451, 10.1007/s11914-021-00688-6. 34117625 PMC8867538

[78] G. Mariño , M. Niso‐Santano , E. H. Baehrecke , and G. Kroemer , “Self‐Consumption: The Interplay of Autophagy and Apoptosis,” Nature Reviews. Molecular Cell Biology 15, no. 2 (2014): 81–94, 10.1038/nrm3735. 24401948 PMC3970201

[79] M. Yang , X. Wei , X. Yi , and D. S. Jiang , “Mitophagy‐Related Regulated Cell Death: Molecular Mechanisms and Disease Implications,” Cell Death & Disease 15, no. 7 (2024): 505, 10.1038/s41419-024-06804-5. 39013891 PMC11252137

[80] M. A. Yapryntseva , B. Zhivotovsky , and V. Gogvadze , “Permeabilization of the Outer Mitochondrial Membrane: Mechanisms and Consequences,” Biochimica et Biophysica Acta ‐ Molecular Basis of Disease 1870, no. 7 (2024): 167317, 10.1016/j.bbadis.2024.167317. 38909847

[81] X. Huang , H. Yan , Z. Xu , B. Yang , P. Luo , and Q. He , “The Inducible Role of Autophagy in Cell Death: Emerging Evidence and Future Perspectives,” Cell Communication and Signaling: CCS 23, no. 1 (2025): 151, 10.1186/s12964-025-02135-w. 40140912 PMC11948861

