# Supplementary material for: Tunnelling Nanotube‐Mediated Lysosome Sharing Promotes Osteocyte Survival via Transcellular Autophagy
Source: Cell Prolif. 2026 May 14;59(7):e70226. doi: 10.1111/cpr.70226 (PMC13325970; doi:10.1111/cpr.70226)
Supplement: Supplementary file 1 — Figure S1: Identification of primary osteocytes from mice and humans. (A) Representative ALP staining images of mouse primary osteoblasts (digest 5) and osteocytes (digest 9). Scale bar, 20 μm. (B) Relative mRNA expression levels of osteocyte‐specific markers E11 and Dmp1 in mouse primary osteoblasts (digest 5), and osteocytes (digest 9) (n = 3). (C) Representative ALP staining images of human primary osteoblasts (digest 5) and osteocytes (digest 9). Scale bar, 20 μm. (D) Relative mRNA expression levels of osteocyte‐specific markers E11 and DMP1 in human primary osteoblasts (digest 5) and osteocytes (digest 9) (n = 3). Data are presented as mean ± SE. Statistical significance was assessed using an unpaired two‐tailed Student's t‐test. **p < 0.01, ***p < 0.001. Figure S2: Effects of cytochalasin B and oxidative stress on cell viability. (A) Effects of cytochalasin B at different concentrations (0, 0.25, 0.5 and 1 μM) on cell viability (n = 3). (B) Effects of 100 and 200 μM H2O2 treatment for 2 h on cell viability (n = 3). (C) Effects of treatment with 100 or 200 μM H2O2 for 24 h on cell viability (n = 3). Data are presented as mean ± SE. Statistical significance was assessed using one‐way ANOVA with Tukey's post hoc test. Figure S3: TNT‐mediated transport of membrane‐bound cargo between MLO‐Y4 cells. Live‐cell imaging of the transport of DiD‐labelled membrane‐bound cargo between MLO‐Y4 cells via TNTs. Arrows indicate DiD‐labelled cargo moving along a TNT. Scale bar, 5 μm. Figure S4: Lysosome transport via TNTs may rescue dying osteocytes. Representative CLSM image of lysosome transport via TNTs between normal and dying osteocytes. White arrows indicate dying osteocytes and yellow arrows indicate lysosomes transported within TNTs. Scale bar, 20 μm. Figure S5: Mitochondrial transfer via TNTs does not restore autophagic flux or prevent apoptosis in osteocytes. (A) Schematic diagram of the co‐culture and FACS strategy using normally cultured MitoTracker‐labelled donor ce [file CPR-59-e70226-s002.docx]

Supporting Information

**Tunnelling Nanotube-Mediated Lysosome Sharing Promotes Osteocyte Survival via Transcellular Autophagy**

Jinbiao Qiang^1,2,3^ | Ronghao Jin^1,2,3^ | Tong Sha^4^ | Fang Zheng^1,2,3^ | Yijun Zhou^5^ | Yue Hu^6^ | Shuyu Zhang^7^ | Zhenming Yang^8^ | Mengdong Nie^1,2,3^ | Huanyu Luo^1,2,3^ | Xiaoduo Tang^1,2,3^ | Hao Guo^1,2,3^ | Zunxuan Xie^1,2,3^ | Jinwei Li^1,2,3^ | Hongchen Sun^1,2,3^ | Cangwei Liu^1,2,3^ | Ce Shi^1,2,3^

^1^Department of Oral Pathology, Hospital of Stomatology, Jilin University, Changchun, China | ^2^Jilin Provincial Key Laboratory of Oral and Craniofacial Diseases & Tissue Reconstruction, Changchun, China | ^3^Key Laboratory of Pathobiology, Ministry of Education, Jilin University, Changchun, China | ^4^Department of Oral and Maxillofacial Surgery, Hospital of Stomatology, Jilin University, Changchun, China | ^5^Department of Endodontics, Hospital of Stomatology, Jilin University, Changchun, China | ^6^School and Hospital of Stomatology, China Medical University, Shenyang, China | ^7^Department of Stomatology, Puyang Oilfield General Hospital, Puyang, China | ^8^Department of Stomatology, Puyang People’s Hospital, Puyang, China

**Correspondence:** Cangwei Liu ([liucw@jlu.edu.cn](mailto:liucw@jlu.edu.cn)) | Ce Shi ([ceshi@jlu.edu.cn](mailto:ceshi@jlu.edu.cn))


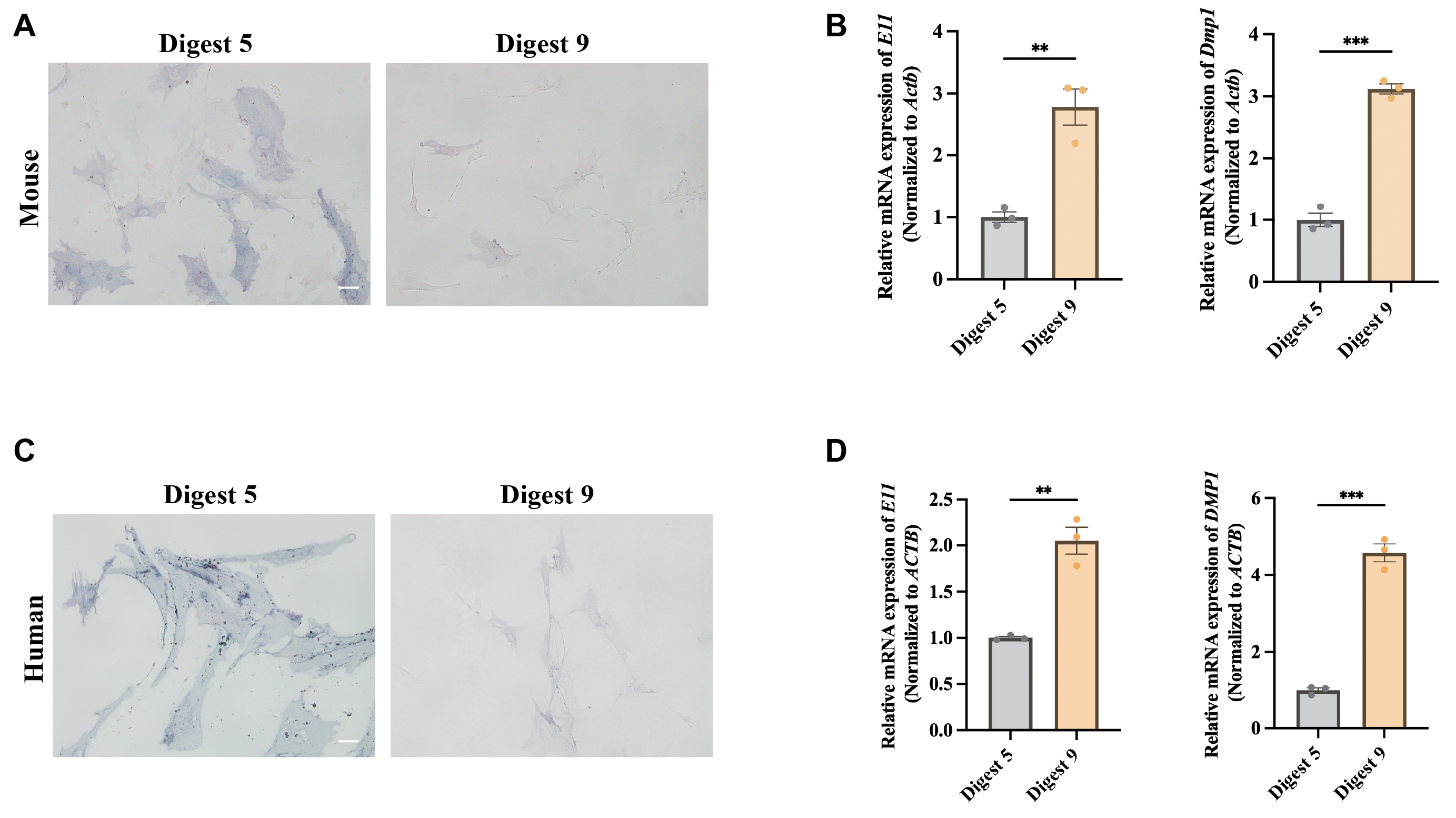


**Figure S1. Identification of primary osteocytes from mice and humans.** A) Representative ALP staining images of mouse primary osteoblasts (digest 5) and osteocytes (digest 9). Scale bar, 20 μm. B) Relative mRNA expression levels of osteocyte-specific markers *E11* and *Dmp1* in mouse primary osteoblasts (digest 5), and osteocytes (digest 9) (*n* = 3). C) Representative ALP staining images of human primary osteoblasts (digest 5) and osteocytes (digest 9). Scale bar, 20 μm. D) Relative mRNA expression levels of osteocyte-specific markers *E11* and *DMP1* in human primary osteoblasts (digest 5) and osteocytes (digest 9) (*n* = 3). Data are presented as mean ± SE. Statistical significance was assessed using an unpaired two-tailed Student’s t-test. ** *p* < 0.01, *** *p* < 0.001.


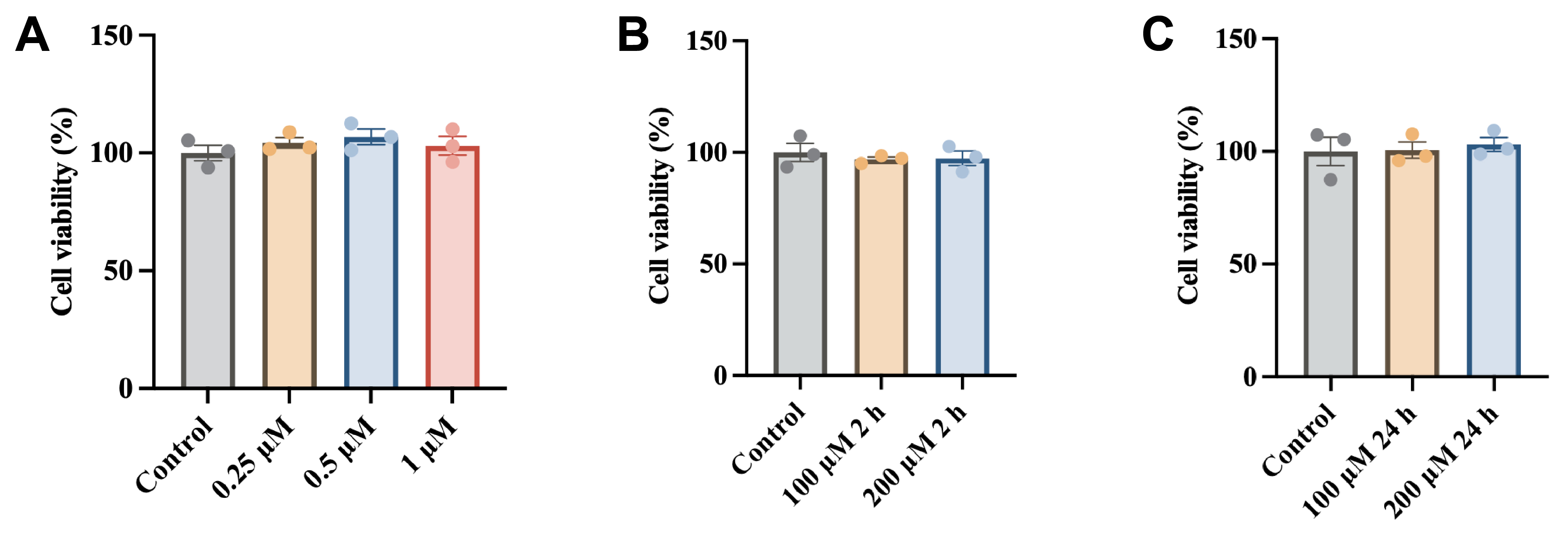


**Figure S2. Effects of cytochalasin B and oxidative stress on cell viability.** A) Effects of cytochalasin B at different concentrations (0, 0.25, 0.5, and 1 μM) on cell viability (*n* = 3). B) Effects of 100 and 200 μM H₂O₂ treatment for 2 h on cell viability (*n* = 3). C) Effects of treatment with 100 or 200 μM H₂O₂ for 24 h on cell viability (*n* = 3). Data are presented as mean ± SE. Statistical significance was assessed using one-way ANOVA with Tukey’s post hoc test.


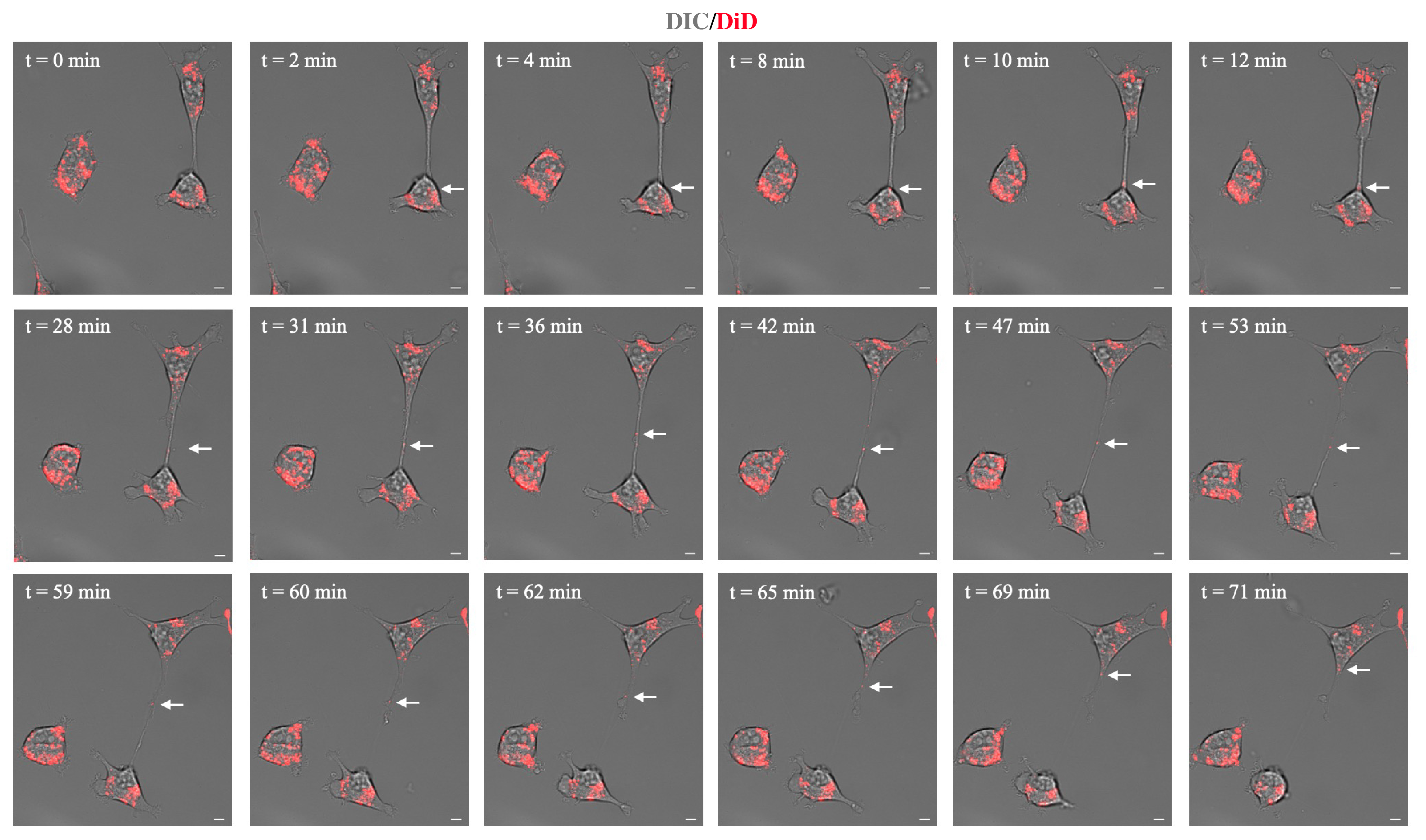


**Figure S3. TNT-mediated transport of** membrane-bound cargo **between MLO-Y4 cells.** Live-cell imaging of the transport of DiD-labelled membrane-bound cargo between MLO-Y4 cells via TNTs. Arrows indicate DiD-labelled cargo moving along a TNT. Scale bar, 5 μm.

**
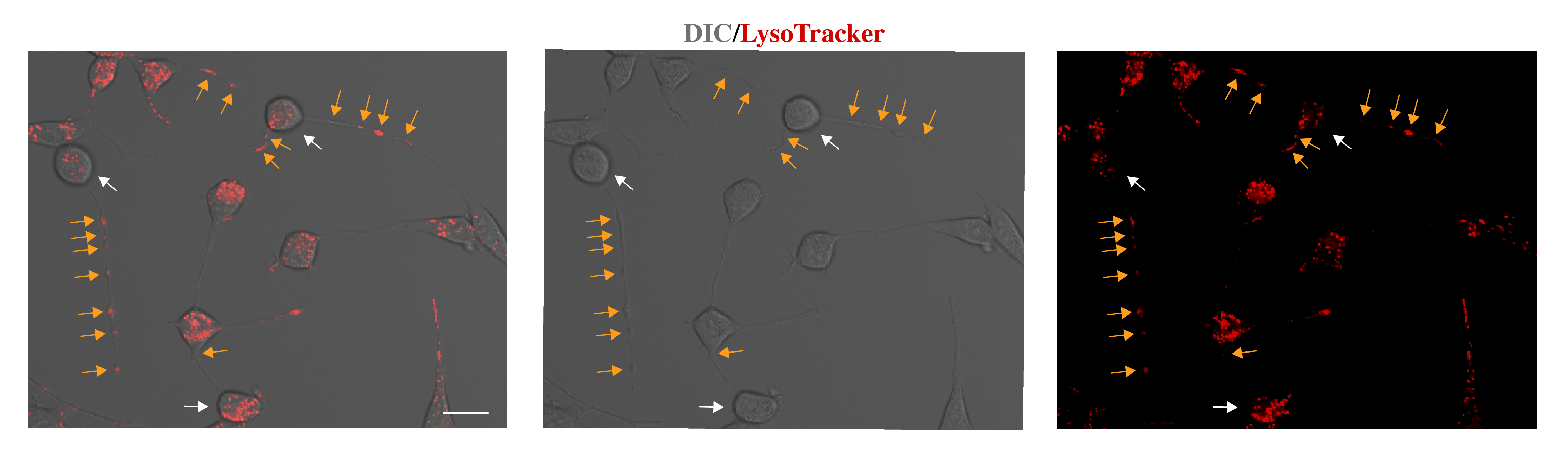
**

**Figure S4. Lysosome transport via TNTs may rescue dying osteocytes.** Representative CLSM image of lysosome transport via TNTs between normal and dying osteocytes. White arrows indicate dying osteocytes and yellow arrows indicate lysosomes transported within TNTs. Scale bar, 20 μm.

**
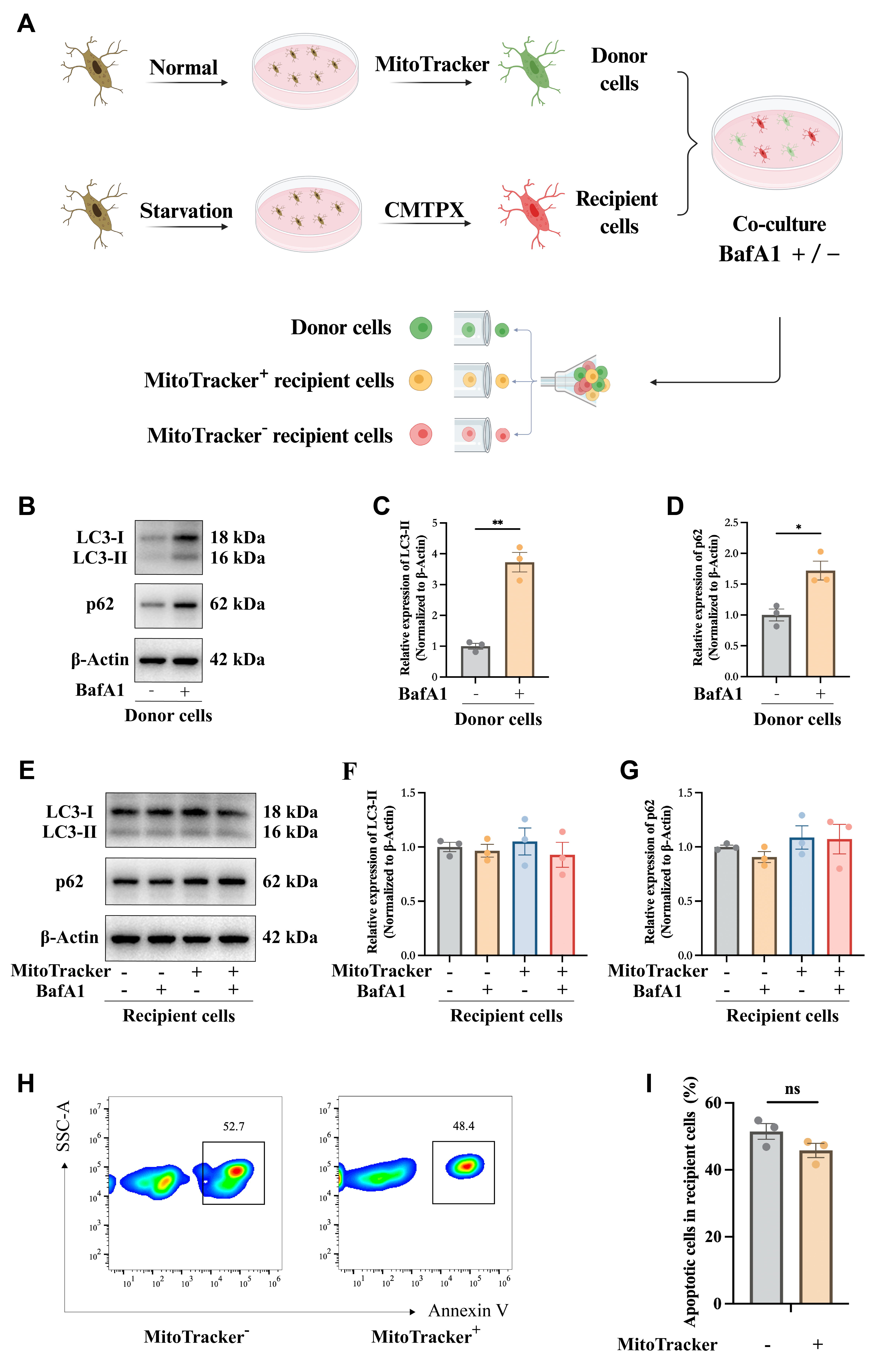
**

**Figure S5. Mitochondrial transfer via TNTs does not restore autophagic flux or prevent apoptosis in osteocytes.** A) Schematic diagram of the co-culture and FACS strategy using normally cultured MitoTracker-labelled donor cells (green) co-cultured with CMTPX-labelled recipient cells (red) under serum starvation, with or without BafA1 treatment. FACS gates: CMTPX⁻/MitoTracker⁺ cells (donor cells), CMTPX⁺/MitoTracker⁺ cells (MitoTracker⁺ recipient cells), and CMTPX⁺/MitoTracker⁻ cells (MitoTracker⁻ recipient cells). B) Western blot analysis of autophagy-related proteins in donor cells after FACS. C, D) Semi-quantification of LC3-II (C) and p62 (D) protein levels in donor cells (*n* = 3). E) Western blot analysis of autophagy-related proteins in recipient cells after FACS. F, G) Semi-quantification of LC3-II (F) and p62 (G) protein levels in recipient cells (*n* = 3). H, I) Flow cytometry plots of Annexin V signals in recipient cells (H) and quantification of the percentage of Annexin V-positive cells in recipient cells (I) (*n* = 3). Data are presented as mean ± SE. Statistical significance was assessed using one-way ANOVA with Tukey’s post hoc test for multiple group comparisons, and an unpaired two-tailed Student’s t-test for two-group comparisons. * *p* < 0.05, ** *p* < 0.01; ns, not significant.

**Movie S1.** **Time-lapse recording of DiD-labelled** membrane-bound cargo **transported between MLO-Y4 osteocytes via TNTs.**

**Table S1.** Primer sequences for qRT-PCR

| Species | Gene Name | Primer Sequence (5′–3′) |
| --- | --- | --- |
| Mouse | *E11* | F: GGACCGTGCCAGTGTTGTTCTG |
|  |  | R: ACCATGCCGTCTCCTGTACCTG |
|  | *Dmp1* | F: CCAGATAACACAAGTCAGGCAGGAG |
|  |  | R: AGGCTGAGGCTCTCGTTGGAC |
|  | *Actb* | F: TGGAATCCTGTGGCATCCATGAAAC |
|  |  | R: TAAAACGCAGCTCAGTAACAGTCCG |
| Human | *E11* | F: GCCAGGTGCCGAAGATGATGTG |
|  |  | R: TGTTGACACTTGTTGCCACCAGAG |
|  | *DMP1* | F: AGCAGTGAGTCCAGCCAAGAGG |
|  |  | R: AGTTGTGGGGTCGGGGTTATCTC |
|  | *ACTB* | F: CTCCATCCTGGCCTCGCTGT |
|  |  | R: GCTGTCACCTTCACCGTTCC |
